# An Immune Response Network Associated with Blood Lipid Levels

**DOI:** 10.1371/journal.pgen.1001113

**Published:** 2010-09-09

**Authors:** Michael Inouye, Kaisa Silander, Eija Hamalainen, Veikko Salomaa, Kennet Harald, Pekka Jousilahti, Satu Männistö, Johan G. Eriksson, Janna Saarela, Samuli Ripatti, Markus Perola, Gert-Jan B. van Ommen, Marja-Riitta Taskinen, Aarno Palotie, Emmanouil T. Dermitzakis, Leena Peltonen

**Affiliations:** 1 Department of Human Genetics, Wellcome Trust Sanger Institute, Wellcome Trust Genome Campus, Hinxton, United Kingdom; 2 Department of Human Genetics, Leiden University Medical Centre, Leiden, The Netherlands; 3 Institute of Molecular Medicine FIMM, University of Helsinki and Unit of Public Health Genomics, National Institute for Health and Welfare, Helsinki, Finland; 4 Unit of Chronic Disease Epidemiology and Prevention, National Institute for Health and Welfare, Helsinki, Finland; 5 Department of General Practice and Primary Health Care, University of Helsinki, Helsinki, Finland; 6 Helsinki University Central Hospital, Unit of General Practice, Helsinki, Finland; 7 Vasa Central Hospital, Vasa, Finland; 8 Folkhälsan Research Centre, Helsinki, Finland; 9 Institute of Molecular Medicine FIMM Technology Center, University of Helsinki, Helsinki, Finland; 10 Department of Medicine, Helsinki University Hospital, University of Helsinki, Helsinki, Finland; 11 The Broad Institute of MIT and Harvard, Cambridge, Massachusetts, United States of America; 12 Department of Clinical Genetics, University of Helsinki and Helsinki University Hospital, Helsinki, Finland; 13 Department of Genetic Medicine and Development, University of Geneva Medical School, Geneva, Switzerland; Stanford University, United States of America

## Abstract

While recent scans for genetic variation associated with human disease have been immensely successful in uncovering large numbers of loci, far fewer studies have focused on the underlying pathways of disease pathogenesis. Many loci which are associated with disease and complex phenotypes map to non-coding, regulatory regions of the genome, indicating that modulation of gene transcription plays a key role. Thus, this study generated genome-wide profiles of both genetic and transcriptional variation from the total blood extracts of over 500 randomly-selected, unrelated individuals. Using measurements of blood lipids, key players in the progression of atherosclerosis, three levels of biological information are integrated in order to investigate the interactions between circulating leukocytes and proximal lipid compounds. Pair-wise correlations between gene expression and lipid concentration indicate a prominent role for basophil granulocytes and mast cells, cell types central to powerful allergic and inflammatory responses. Network analysis of gene co-expression showed that the top associations function as part of a single, previously unknown gene module, the Lipid Leukocyte (LL) module. This module replicated in T cells from an independent cohort while also displaying potential tissue specificity. Further, genetic variation driving LL module expression included the single nucleotide polymorphism (SNP) most strongly associated with serum immunoglobulin E (IgE) levels, a key antibody in allergy. Structural Equation Modeling (SEM) indicated that LL module is at least partially reactive to blood lipid levels. Taken together, this study uncovers a gene network linking blood lipids and circulating cell types and offers insight into the hypothesis that the inflammatory response plays a prominent role in metabolism and the potential control of atherogenesis.

## Author Summary

Circulating lipid concentrations are important predictors of coronary artery disease. The main pathology of coronary artery disease is atherosclerosis, a cycle of lipid adherence to the walls of arteries and an inflammatory response resulting in more adhesion. To investigate the link between lipids and immune cells in circulation, we have generated both genomic and whole blood gene expression profiles for a population-based collection of individuals from the capital region of Finland. Key mediators of inflammation and allergy were shown to be correlated with lipid levels. Further, the expressions of these genes operated in such a highly coordinated fashion that they appeared to function as part of a single pathway, which itself was both highly correlated with and reactive to lipid levels. Our findings offer insight into how lipids activate circulating immune cells, potentially contributing to the pathogenesis of coronary artery disease.

## Introduction

Blood lipid levels have long been known to be important markers of coronary artery disease and the underlying pathology of atherosclerosis [1], [2]. High-density lipoprotein cholesterol (HDL) is a small, dense complex of phospholipids and apolipoproteins, including apolipoprotein A1 (APOA1), which is synthesized in the liver and has been shown to be negatively associated with atherogenesis. Low-density lipoprotein cholesterol (LDL) displays a positive association with atherogenesis and contains one apolipoprotein, apolipoprotein B (APOB), as well as numerous fatty acids, lipids, and cholesterols. Atherosclerosis entails the buildup of LDL deposits in the arterial wall where they undergo oxidation and subsequent internalization by macrophages, an inflammatory response, leading to the formation of foam cells and further inflammatory signals which can exacerbate arterial LDL adhesion, leading to stenosis [3].

Genome-wide association studies (GWAS) have yielded many successes in the search for the common genetic variants underlying blood lipid levels and other metabolic traits [4]–[7], however systematic functional investigation of pathways, particularly lipid pathways, has lagged behind. Recently, the link between the inflammatory response and metabolism has been the subject of intense research [8], [9]. Chronic inflammation has been shown to lead to the activation of c-Jun amino-terminal kinases [10], [11], and plasma triglyceride levels have been associated with various mediators of NF-ΚB, a key component of the immune response [12]–[15]. Further, it has been shown that postprandial triglyceride increase activates monocytes and neutrophils and the cardioprotective properties of HDL might be partially mediated by activation of the complement cascade [16], [17]. Recently, two companion studies demonstrated both an enrichment of immune pathways in metabolic syndrome and the utility of integrating genomic and transcriptional variation [18], [19]. In particular, they identify a gene expression network of macrophage origin which is likely to be causative of various metabolic traits.

The proximity of lipid compounds and leukocytes in peripheral blood offers a uniquely accessible system in which to study their interactions. We utilized total blood samples from a population-based cohort of 518 unrelated individuals (240 males and 278 females, aged 25–74 years) from the Dietary, Lifestyle, and Genetic determinants of Obesity and Metabolic syndrome (DILGOM) study which have undergone both genome-wide expression profiling and genome-wide genotyping with imputation. After quality filtering (**Materials and Methods**), 35,419 expression probes and 541,654 SNPs (2,061,516 SNPs after imputation) were taken forward for further analyses. We first assessed how single gene expression correlated with both the specific lipid measurements of the DILGOM cohort and overall variation in lipids (**Table 1**) then performed network analyses to identify and characterize clusters of tightly co-expressed genes, modules, which showed strong association with lipids. Replication and tissue specificity of a particular module, termed the Lipid Leukocyte (LL) module, was investigated in an independent cohort of B cells and T cells. Finally, genetic variation was assessed both to identify expression quantitative trait loci (eQTLs) driving expression of LL module, thus connecting our findings with that of a previously published GWAS, and to construct an edge-oriented network to elucidate the chain of causality.

**Table 1:** Lipid traits of the DILGOM population sample.

Trait	Units	Overall mean (s.d.)	Male mean (s.d.)	Female mean (s.d.)
TC	mmol/L	5.11 (0.95)	5.09 (0.95)	5.13 (0.95)
LDL	mmol/L	3.07 (0.84)	3.17 (0.84)	2.98 (0.83)
HDL	mmol/L	1.48 (0.36)	1.34 (0.30)	1.61 (0.36)
APOA1	g/L	1.63 (0.28)	1.53 (0.25)	1.72 (0.28)
APOB	g/L	0.92 (0.21)	0.95 (0.21)	0.89 (0.20)
TG	mmol/L	1.15 (0.64)	1.27 (0.78)	1.04 (0.47)
FFA	mmol/L	0.36 (0.21)	0.33 (0.20)	0.38 (0.22)

## Results and Discussion

To assess how each lipid trait associated with gene expression, levels of HDL, LDL, APOB, APOA1, total serum cholesterol (TC), triglycerides (TG), and free fatty acids (FFA) were modeled using multiple linear regression with appropriate covariates (**Table 2**) and a Bonferroni adjusted significance level for each trait, equivalent to a nominal *P* = 1.41×10^−6^. Models were fitted with and without hypertension and cholesterol lowering medications as covariates; no difference in the results was found. Since there are known gender-specific effects, traits were gender-stratified and standardized to *Z*-scores. Overall, 49 significant associations with gene expression were found (**Table 3**), however none were observed for TC, LDL, or APOA1. All reported *P* values are Bonferroni adjusted.

**Table 2:** Multiple regression covariates.

TRAIT	Abbrev.	COVARIATES			
Total serum cholesterol	TC	Age	CM	HTM	
High density lipoprotein	HDL	Age	CM	HTM	Alcohol
Low density lipoprotein	LDL	Age	CM	HTM	Alcohol
Apolipoprotein A1	APOA1	Age	CM	HTM	
Apolipoprotein B	APOB	Age	CM	HTM	
Triglycerides	TG	Age	CM	HTM	Alcohol
Free fatty acids	FFA	Age	CM	HTM	
Meta-lipids	-	Age	CM	HTM	Alcohol

CM = cholesterol lowering medication (Yes or No).

HTM = hypertension medication (Yes or No).

Alcohol = alcohol intake in previous 7 days (grams).

For those DILGOM individuals passing quality control (N = 518), the proportion using HTM was 20.5% (18.7% of females, 22.5% of males) and the proportion using CM was 14.3% (10.1% of females, 19.2% of males).

**Table 3:** Genes showing significant evidence of association with specific lipid traits.

Trait	Chromosome	Position	*P* value	*P* value (corrected)	Beta (95% CI)	Gene
APOB	15	48321493	9.90E-10	3.51E-05	−0.39 (−0.51–−0.27)	*HDC*
APOB	3	129681589	6.11E-08	2.16E-03	−0.62 (−0.84–−0.40)	*GATA2*
APOB	3	150097094	1.54E-07	5.45E-03	−0.53 (−0.73–−0.34)	*CPA3*
APOB	1	204405726	1.96E-07	6.96E-03	−0.93 (−1.27–−0.58)	*C1ORF186*
APOB	1	203893823	2.51E-07	8.90E-03	−0.61 (−0.84–−0.38)	*SLC45A3*
APOB	1	157544285	3.83E-07	1.36E-02	−0.34 (−0.47–−0.21)	*FCER1A*
HDL	15	48321493	5.94E-08	2.11E-03	0.35 (0.23–0.48)	*HDC*
HDL	1	203893823	5.36E-07	1.90E-02	0.60 (0.37–0.83)	*SLC45A3*
HDL	1	157544285	8.56E-07	3.03E-02	0.33 (0.20–0.46)	*FCER1A*
FFA	3	48869652	1.02E-41	3.63E-37	2.85 (2.47–3.22)	*SLC25A20*
FFA	7	95051149	1.56E-25	5.51E-21	1.41 (1.16–1.66)	*PDK4*
FFA	11	68283656	2.97E-13	1.05E-08	2.32 (1.71–2.93)	*CPT1A*
FFA	9	19106194	1.34E-08	4.74E-04	0.86 (0.57–1.15)	*ADFP*
FFA	9	19106260	1.35E-08	4.79E-04	1.25 (0.83–1.67)	*ADFP*
FFA	4	159847295	1.94E-08	6.88E-04	1.45 (0.95–1.95)	*ETFDH*
TG	15	48321493	2.46E-37	8.71E-33	−0.79 (−0.90–−0.68)	*HDC*
TG	3	150097094	1.47E-31	5.22E-27	−1.15 (−1.33–−0.97)	*CPA3*
TG	1	203893823	1.69E-28	5.97E-24	−1.28 (−1.50–−1.07)	*SLC45A3*
TG	3	129681589	2.22E-28	7.88E-24	−1.24 (−1.45–−1.04)	*GATA2*
TG	11	59622246	2.09E-27	7.40E-23	−1.62 (−1.90–−1.35)	*MS4A2*
TG	1	157544285	1.11E-26	3.92E-22	−0.70 (−0.82–−0.58)	*FCER1A*
TG	8	33490552	8.93E-22	3.16E-17	0.81 (0.65–0.97)	*SNORD13*
TG	11	55415610	1.06E-21	3.74E-17	−1.27 (−1.51–−1.02)	*SPRYD5*
TG	1	204405726	3.06E-20	1.08E-15	−1.63 (−1.97–−1.30)	*C1ORF186*
TG	6	26212414	3.32E-18	1.17E-13	0.80 (0.62–0.97)	*HIST1H4C*
TG	11	59594827	1.07E-15	3.79E-11	−0.78 (−0.96–−0.59)	*MS4A3*
TG	21	29469766	6.16E-12	2.18E-07	−0.67 (−0.86–−0.49)	*C21ORF7*
TG	1	245806385	4.76E-11	1.69E-06	−2.10 (−2.71–−1.49)	*C1ORF150*
TG	1	45014767	9.99E-11	3.54E-06	2.51 (1.77–3.26)	*SNORD46*
TG	12	115953221	1.16E-10	4.09E-06	1.36 (0.96–1.77)	*FBXW8*
TG	19	50688370	3.26E-10	1.16E-05	0.96 (0.67–1.26)	*RTN2*
TG	6	16256091	1.06E-09	3.74E-05	−1.22 (−1.61–−0.84)	*MYLIP*
TG	9	89330079	4.45E-09	1.58E-04	0.34 (0.23–0.46)	*DAPK1*
TG	9	106583321	4.93E-09	1.74E-04	−0.61 (−0.81–−0.41)	*ABCA1*
TG	1	152230698	5.35E-09	1.90E-04	0.41 (0.28–0.55)	*RPS27*
TG	6	34339344	2.76E-08	9.76E-04	0.53 (0.34–0.71)	
TG	6	132103151	5.03E-08	1.78E-03	−1.74 (−2.36–−1.13)	*ENPP3*
TG	5	85949563	5.95E-08	2.11E-03	0.37 (0.24–0.50)	*COX7C*
TG	7	134500907	6.42E-08	2.27E-03	−0.59 (−0.79–−0.38)	*TMEM140*
TG	19	16129952	8.98E-08	3.18E-03	−0.74 (−1.01–−0.47)	*HSH2D*
TG	1	190421142	2.27E-07	8.05E-03	−0.53 (−0.72–−0.33)	*RGS18*
TG	11	54794809	2.72E-07	9.64E-03	−1.92 (−2.64–−1.20)	*TRIM48*
TG	1	154248226	3.65E-07	1.29E-02	1.14 (0.71–1.57)	*SSR2*
TG	22	17803280	4.35E-07	1.54E-02	1.35 (0.83–1.87)	*MRPL40*
TG	12	87929168	7.78E-07	2.75E-02	−1.96 (−2.73–−1.19)	*HS.132563*
TG	7	65709374	8.08E-07	2.86E-02	0.47 (0.28–0.65)	
TG	1	26519273	9.00E-07	3.19E-02	0.38 (0.23–0.53)	*CD52*
TG	1	235211299	9.25E-07	3.28E-02	0.42 (0.26–0.59)	
TG	8	48812282	1.29E-06	4.57E-02	−0.64 (−0.89–−0.38)	*CEBPD*
TG	2	192407481	1.36E-06	4.82E-02	−0.47 (−0.65–−0.28)	*SDPR*

### Mediators of inflammation and allergy are associated with APOB, HDL, and TG levels

The strongest signals for FFA were from genes previously known to be involved in β-oxidation and lipolysis. During fatty acid metabolism, long-chain acyl groups are transported from the cytosol into the mitochondrial matrix by carnitine. At the outer mitochondrial membrane, acyl groups are attached to carnitine by carnitine palmitoyltransferase 1A (*CPTA1*, *P* = 1.05×10^−8^ for FFA) and internalized by carnitine/acylcarnitine translocase (*SLC25A20*, *P* = 3.63×10^−37^) [20]. Pyruvate dehydrogenase kinase 4 (*PDK4*, *P* = 5.51×10^−21^) resides in the mitochondrial matrix and downregulates the activity of the pyruvate dehydrogenase complex, a process important to the substrate competition between fatty acids and glucose [21]. Further, two strongly associated probes lie within adipose differentiation-related protein (*ADFP*, *P* = 4.74×10^−4^ and *P* = 4.79×10^−4^) which encodes adipophilin [22], [23], and electron-transferring-flavoprotein dehydrogenase (*ETFDH*, *P* = 6.88×10^−4^) has been previously linked to multiple acyl-CoA dehydrogenation deficiency disorders [24].

While lipid traits were correlated with each other (**Figure S1**), it was of particular interest that the top associations across APOB, HDL and TG were largely shared (**Table 3**). These genes included histidine decarboxylase (*HDC*), the alpha subunit from the Fc fragment of high affinity IgE receptor (*FCER1A*), prostein (*SLC45A3*), GATA binding protein 2 (*GATA2*), and carboxypeptidase A3 (*CPA3*). These genes were also significant predictors of the APOB-APOA1 ratio, the strongest cholesterol-based risk factor for atherosclerosis and coronary artery disease [25] (**Table 4**). Differences in transcript levels between samples can arise from the relative expansion or contraction of cell populations, thus to test whether the associations could be due to variation in the relative abundance of a range of blood cell types, previously identified cell type expression markers [26] were added as covariates in the model; significance was unchanged (**Table 5**). Given inter-trait correlations, multivariate approaches may offer better power to detect relationships between lipids and gene expression by incorporating information from cross-trait covariance [27], [28] (**Materials and Methods**). When predicting multiple traits simultaneously (termed meta-lipids), 85 unique associations were observed at an equivalent Bonferroni-corrected significance level and the above genes remained strongly associated (**Table S1**). This represented an almost two-fold increase in the number of significant associations using single lipid traits and offered a unified ranking for assessing each gene's involvement in lipid levels.

**Table 4:** *HDC*, *FCER1A*, *CPA3*, *SLC45A3*, and *GATA2* show significant association with the APOB/APOA1 ratio.

Trait	Chromosome	Position	*P* value	*P* value (corrected)	Beta (95% CI)	Gene
APOB/APOA1	15	48321493	3.17E-09	1.12E-04	−0.39 (−0.51–−0.26)	*HDC*
APOB/APOA1	1	157544285	7.28E-08	2.58E-03	−0.37 (−0.50–−0.23)	*FCER1A*
APOB/APOA1	1	203893823	2.29E-07	8.11E-03	−0.63 (−0.86–−0.39)	*SLC45A3*
APOB/APOA1	3	150097094	5.12E-07	1.81E-02	−0.52 (−0.72–−0.32)	*CPA3*
APOB/APOA1	3	129681589	5.40E-07	1.91E-02	−0.59 (−0.82–−0.36)	*GATA2*

**Table 5:** Significance of *HDC*, *FCER1A*, *CPA3*, *SLC45A3*, and *GATA2* with cell-type specific expression markers as covariates.

Trait	Chromosome	Position	*P* value	*P* value (corrected)	Beta (95% CI)	Gene
APOB	15	48321493	1.62E-09	5.74E-05	−0.40 (−0.53–−0.28)	*HDC*
APOB	3	150097094	6.71E-08	2.38E-03	−0.58 (−0.79–−0.37)	*CPA3*
APOB	1	157544285	9.24E-08	3.27E-03	−0.38 (−0.52–−0.24)	*FCER1A*
APOB	1	203893823	1.72E-07	6.11E-03	−0.66 (−0.90–−0.41)	*SLC45A3*
APOB	3	129681589	5.58E-07	1.98E-02	−0.60 (−0.84–−0.37)	*GATA2*
HDL	15	48321493	5.13E-07	1.82E-02	0.34 (0.21–0.46)	*HDC*
HDL	1	157544285	5.81E-07	2.06E-02	0.35 (0.22–0.49)	*FCER1A*
HDL	1	203893823	1.34E-06	4.75E-02	0.60 (0.36–0.84)	*SLC45A3*
FFA	3	48869652	2.11E-39	7.48E-35	2.93 (2.53–3.33)	*SLC25A20*
FFA	7	95051149	1.72E-26	6.10E-22	1.63 (1.35–1.91)	*PDK4*
FFA	11	68283656	8.81E-13	3.12E-08	2.32 (1.70–2.94)	*CPT1A*
FFA	9	19106260	1.88E-08	6.66E-04	1.32 (0.87–1.77)	*ADFP*
FFA	9	19106194	2.30E-08	8.14E-04	0.94 (0.62–1.26)	*ADFP*
FFA	4	159847295	3.92E-07	1.39E-02	1.53 (0.95–2.11)	*ETFDH*
TG	15	48321493	3.59E-38	1.27E-33	−0.82 (−0.93–−0.71)	*HDC*
TG	1	157544285	4.05E-32	1.43E-27	−0.80 (−0.93–−0.68)	*FCER1A*
TG	3	150097094	1.58E-31	5.60E-27	−1.20 (−1.38–−1.01)	*CPA3*
TG	1	203893823	1.72E-29	6.10E-25	−1.36 (−1.58–−1.14)	*SLC45A3*
TG	3	129681589	4.16E-28	1.47E-23	−1.27 (−1.49–−1.06)	*GATA2*

The linear models are the same as in Table 1 of the main text, except for the addition of covariates for each of the cell-type specific expression profiles in Whitney et al [26]. These include proportions of lymphocytes, neutrophils, reticulocytes, B cells, cytotoxic T lymphocytes/natural killer cells, erythrocytes, myeloid cells, and Myc-regulated cells (profiles for the time of day were also included). There were no T cell specific markers available on the Illumina HT-12. Covariates were constructed via an average standard score across all cell-type specific markers for each sample.

The most strongly associated genes for APOB, HDL, and TG present intriguing candidate genes for metabolic dysfunction, inflammation, and atherosclerosis. *HDC* encodes the catalyst for the conversion of histidine to histamine, a well-known pro-inflammatory molecule that is secreted by basophils and mast cells (BMCs). Histamine plays a role in atherogenesis and HDC expression has been previously associated with atherosclerotic status [29]. Importantly, lipoproteins, in particular very low-density lipoprotein, have been shown to cause secretion of histamine from basophils [30]. *HDC* may also play a more general role in metabolic syndrome as murine knockouts display hyperleptinemia, obesity, and glucose intolerance [31], [32]. On the cell surfaces of BMCs, FCER1A plays a powerful role in the immune response and in histamine release as the encoded receptor subunit directly interacts with antigen-bound IgE, an antibody isotype capable of the most potent immune reactions [33]. *FCER1A* was also found to be the strongest signal in a recent GWAS of serum IgE levels [34]. Interestingly, biochemical studies of mast cell specific CPA3 have shown its involvement in the degradation of APOB from LDL thus leading to the potential for LDL fusion [35]–[37]. Our observation of a negative correlation between *CPA3* expression and APOB concentrations was consistent with these findings. The transcription factor *GATA2* has been shown to both attenuate inflammation in murine adipose tissue and allow for normal mast cell development [38]. Weidinger et al. previously observed the co-expression of *GATA2* and *FCER1A* [34]. The correlation of *FCER1A* and *GATA2* expression in the DILGOM cohort was also extremely strong (Spearman's ρ = 0.664), therefore we investigated the hypothesis that *HDC*, *FCER1A*, *SLC45A3*, *GATA2*, and *CPA3* function as part of the same pathway.

### Network analysis of gene co-expression and module replication

**Figure 1:**
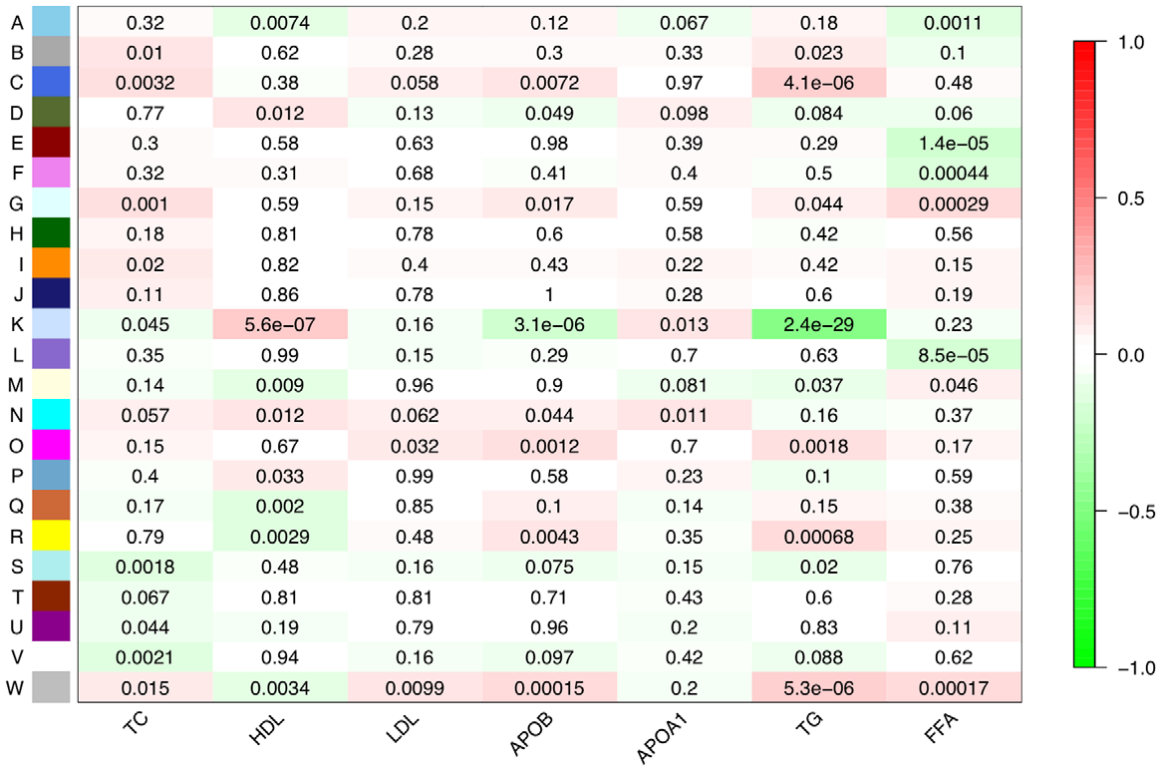
Network module associations with lipid traits. For each lipid trait and each module expression profile, a Spearman rank correlation was performed. Each row corresponds to a module (arbitrarily lettered from A to W) and each column a particular lipid trait. Each cell contains the probability that a correlation exists by chance and is color-coded with red indicating a strong positive correlation and green a strong negative correlation. The module most strongly associated with lipid traits was module K. The genes composing this module, the Lipid Leukocyte (LL) module, show that many of the top signals from a standard linear regression were part of the same sub-network.

**Figure 2:**
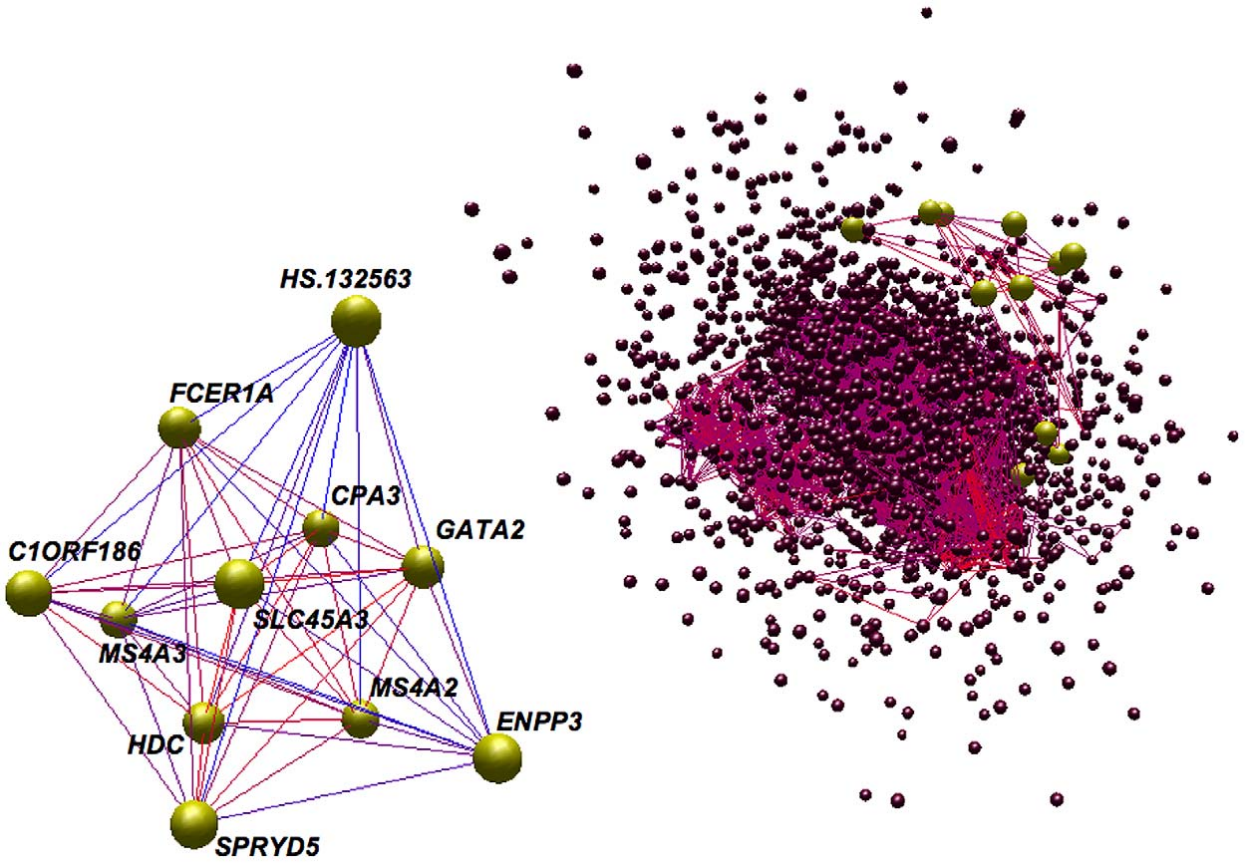
Topology of the network and the LL module. The co-expression patterns of the network and the LL module were rendered using BiolayoutExpress3D [60]. Each node is a gene (node size is not significant) and each edge is colored according to the absolute value of the Pearson correlation between two nodes, red being strong and blue being weak. The LL module has been colored yellow and, within the topology of the network (right panel), has been enlarged relative to other nodes. The topology of the network has been edge filtered (Pearson<0.65) in order to make strong correlations clearer.

**Figure 3:**
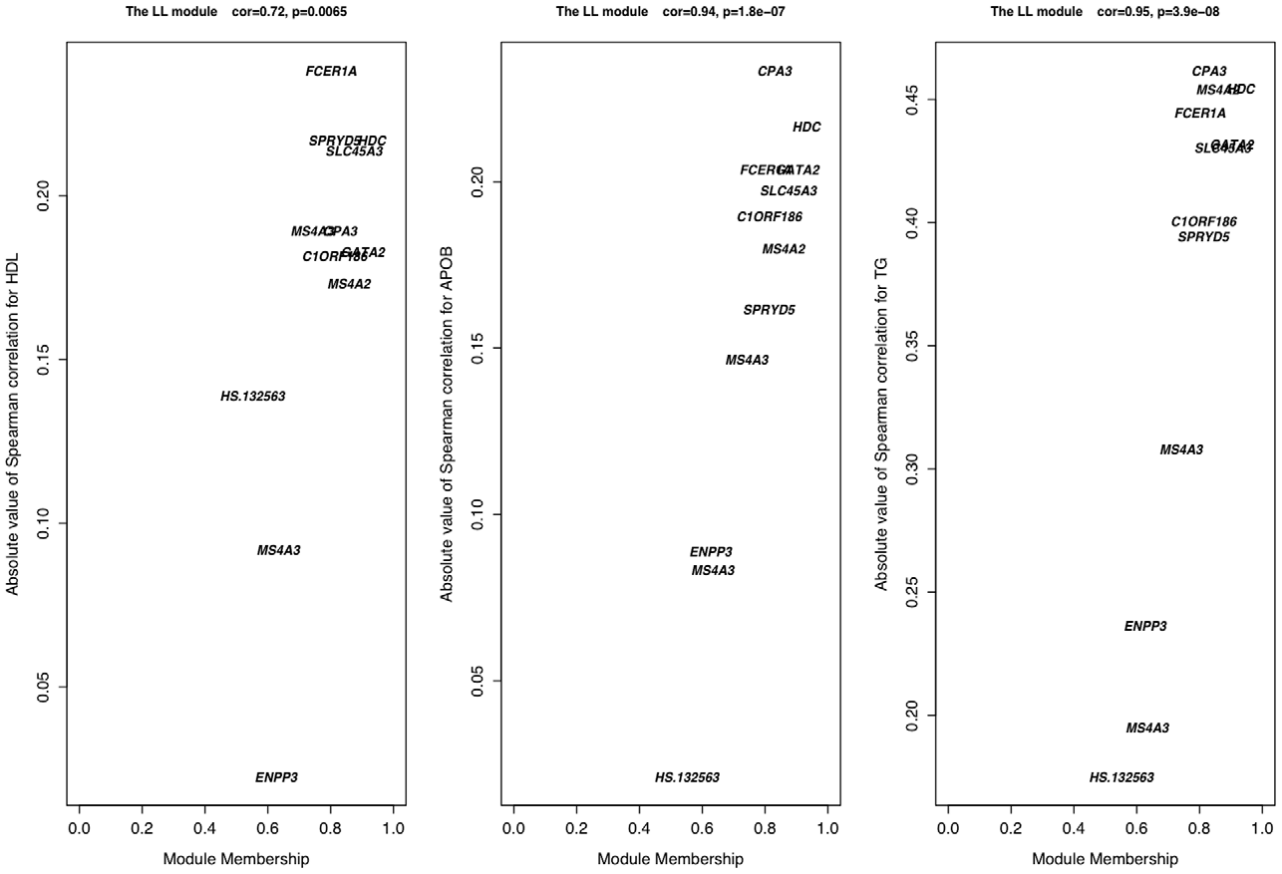
Connectivity and trait association within the LL module. In the LL module, there is a strong positive correlation between the intra-modular connectivity of a node (gene) and its association with APOB, HDL, and TG levels, showing that the approximately eight genes inter-connected are also the most associated with lipid traits. These genes constitute the core of the LL module.

In biological pathways, many genes tend to co-express thus it is natural to incorporate these correlations into a network-based framework. Within this framework, pairwise correlations between genes are used to describe the connectedness of the network, and clusters of tightly correlated genes (modules) can define pathways. To construct a co-expression network that characterizes lipid traits, the method of Horvath and Langfelder [39], [40] was used to assess the top 10% of expression signals for meta-lipids (3,520 unique signals, **Materials and Methods**). Twenty-three modules were identified and each module's summary expression profile (defined by its first principal component) was tested for correlation with individual lipid traits (**Figure 1**). The strongest expression associations identified above for HDL, APOB, and TG clustered into the same pathway, module K, hereafter referred to as the Lipid Leukocyte (LL) module (**Figure 2**). The strongest signals for FFA did not cluster into a module. Summary expression of LL module was associated with HDL (*P* = 5.62×10^−7^), APOB (*P* = 3.06×10^−6^), and TG levels (*P* = 2.44×10^−29^), results which were significant after correcting for the estimated number of co-expression modules in the whole gene set (**Materials and Methods**). It is composed of 11 genes (12 probes) including *HDC*, *FCER1A*, *GATA2*, *CPA3*, *MS4A2* (the beta subunit of high affinity IgE receptor's Fc fragment), *SPRYD5* and *SLC45A3* (**Table S2**). Module membership, a measure of intramodular connectivity, showed that the afore mentioned genes constitute the core of the module and are the most correlated with lipid traits (**Figure 3**).

In order to replicate LL module's existence and investigate tissue specificity, we utilized expression data from the GenCord cohort [41], a unique resource which includes both EBV-immortalized B cell lines (LCLs) and primary T cells drawn from individual umbilical cord blood. LL module co-expression was highly significant in T cells, however LCLs from the same individuals showed a marked absence of any co-expression (**Table 6**). This suggests that this co-expression module is tissue specific among blood cell types, however it is not clear whether, or to what extent, laboratory treatment might also contribute to the obscurity of co-expression networks. The possibility exists that significant changes in host-cell gene expression patterns occur upon both infection of B cells with EBV, which binds to complement receptors thus initiating the complement system, and the selection of B cells which have successfully integrated episomal EBV. We therefore emphasize caution when interpreting correlations in gene expression from non-primary tissues and encourage further studies into the effects of laboratory treatments.

**Table 6:** LL module core gene co-expression in the GenCord cohort.

		DILGOM (whole blood)		GenCord (T cells)		GenChord (LCLs)	
Gene A	Gene B	Spearman rho	*P* value	Spearman rho	*P* value	Spearman rho	*P* value
CPA3	FCER1A	0.701	<2.2E-16	0.917	<2.2E-16	−0.055	0.636235841
CPA3	GATA2	0.705	<2.2E-16	0.917	<2.2E-16	0.035	0.767344119
CPA3	HDC	0.774	<2.2E-16	0.650	<2.2E-16	0.060	0.607446891
CPA3	MS4A2	0.682	<2.2E-16	0.866	<2.2E-16	0.135	0.246666555
CPA3	SLC45A3	0.812	<2.2E-16	0.843	<2.2E-16	0.058	0.618207944
CPA3	SPRYD5	0.619	<2.2E-16	0.160	0.168711659	−0.060	0.607702148
FCER1A	CPA3	0.701	<2.2E-16	0.917	<2.2E-16	−0.055	0.636235841
FCER1A	GATA2	0.657	<2.2E-16	0.858	<2.2E-16	−0.104	0.375296225
FCER1A	HDC	0.684	<2.2E-16	0.576	1.18E-07	−0.115	0.325281824
FCER1A	MS4A2	0.734	<2.2E-16	0.880	<2.2E-16	−0.319	0.005509442
FCER1A	SLC45A3	0.651	<2.2E-16	0.852	<2.2E-16	0.006	0.961262695
FCER1A	SPRYD5	0.605	<2.2E-16	0.237	0.041109859	0.276	0.016997624
GATA2	CPA3	0.705	<2.2E-16	0.917	<2.2E-16	0.035	0.767344119
GATA2	FCER1A	0.657	<2.2E-16	0.858	<2.2E-16	−0.104	0.375296225
GATA2	HDC	0.890	<2.2E-16	0.655	<2.2E-16	0.104	0.371949805
GATA2	MS4A2	0.739	<2.2E-16	0.882	<2.2E-16	−0.009	0.935643163
GATA2	SLC45A3	0.828	<2.2E-16	0.795	<2.2E-16	0.086	0.464335679
GATA2	SPRYD5	0.762	<2.2E-16	0.055	0.638233048	−0.063	0.591125476
HDC	CPA3	0.774	<2.2E-16	0.650	<2.2E-16	0.060	0.607446891
HDC	FCER1A	0.684	<2.2E-16	0.576	1.18E-07	−0.115	0.325281824
HDC	GATA2	0.890	<2.2E-16	0.655	<2.2E-16	0.104	0.371949805
HDC	MS4A2	0.770	<2.2E-16	0.623	3.99E-09	0.020	0.865198439
HDC	SLC45A3	0.856	<2.2E-16	0.599	2.82E-08	−0.277	0.016265791
HDC	SPRYD5	0.791	<2.2E-16	−0.056	0.630865052	−0.171	0.141566903
MS4A2	CPA3	0.682	<2.2E-16	0.866	<2.2E-16	0.135	0.246666555
MS4A2	FCER1A	0.734	<2.2E-16	0.880	<2.2E-16	−0.319	0.005509442
MS4A2	GATA2	0.739	<2.2E-16	0.882	<2.2E-16	−0.009	0.935643163
MS4A2	HDC	0.770	<2.2E-16	0.623	3.99E-09	0.020	0.865198439
MS4A2	SLC45A3	0.718	<2.2E-16	0.783	<2.2E-16	−0.003	0.981884832
MS4A2	SPRYD5	0.674	<2.2E-16	0.199	0.087013944	−0.347	0.002401304
SLC45A3	CPA3	0.812	<2.2E-16	0.843	<2.2E-16	0.058	0.618207944
SLC45A3	FCER1A	0.651	<2.2E-16	0.852	<2.2E-16	0.006	0.961262695
SLC45A3	GATA2	0.828	<2.2E-16	0.795	<2.2E-16	0.086	0.464335679
SLC45A3	HDC	0.856	<2.2E-16	0.599	2.82E-08	−0.277	0.016265791
SLC45A3	MS4A2	0.718	<2.2E-16	0.783	<2.2E-16	−0.003	0.981884832
SLC45A3	SPRYD5	0.758	<2.2E-16	0.202	0.082789037	0.083	0.479958577
SPRYD5	CPA3	0.619	<2.2E-16	0.160	0.168711659	−0.060	0.607702148
SPRYD5	FCER1A	0.605	<2.2E-16	0.237	0.041109859	0.276	0.016997624
SPRYD5	GATA2	0.762	<2.2E-16	0.055	0.638233048	−0.063	0.591125476
SPRYD5	HDC	0.791	<2.2E-16	−0.056	0.630865052	−0.171	0.141566903
SPRYD5	MS4A2	0.674	<2.2E-16	0.199	0.087013944	−0.347	0.002401304
SPRYD5	SLC45A3	0.758	<2.2E-16	0.202	0.082789037	0.083	0.479958577

Each core gene in the LL module was tested for co-expression against all other core genes in the LL module using Spearman's rank correlation coefficient. This was done across three datasets: whole blood extracts from the DILGOM cohort (N = 518), primary T cells from GenChord individuals (N = 75), and EBV-transformed B cells from the same GenCord individuals. Given 21 tests for each cohort, the Bonferroni corrected significance level is 2.38×10^−3^.

### eQTL analysis of the Lipid Leukocyte module

Gene expression itself is a quantitative trait of genetic variation [42]–[44]. Using genome-wide SNP genotypes from individuals in DILGOM, we investigated the genetic effects on expression for each gene in LL module and for the LL module as a whole (**Materials and Methods**). For those SNPs in *cis*, within 1 Mb of the expression probe midpoint, a simple linear regression was performed. In order to determine significance, a permutation procedure was implemented [43]. For *trans* SNPs, greater than 5 Mb away or on a different chromosome, the non-parametric Spearman rank correlation was used [42], offering a more robust test of association since permutation across the whole genome would be computationally prohibitive. To determine the significance of the nominal Spearman *P* value, a threshold of 5.0×10^−7^ was implemented.

**Figure 4:**
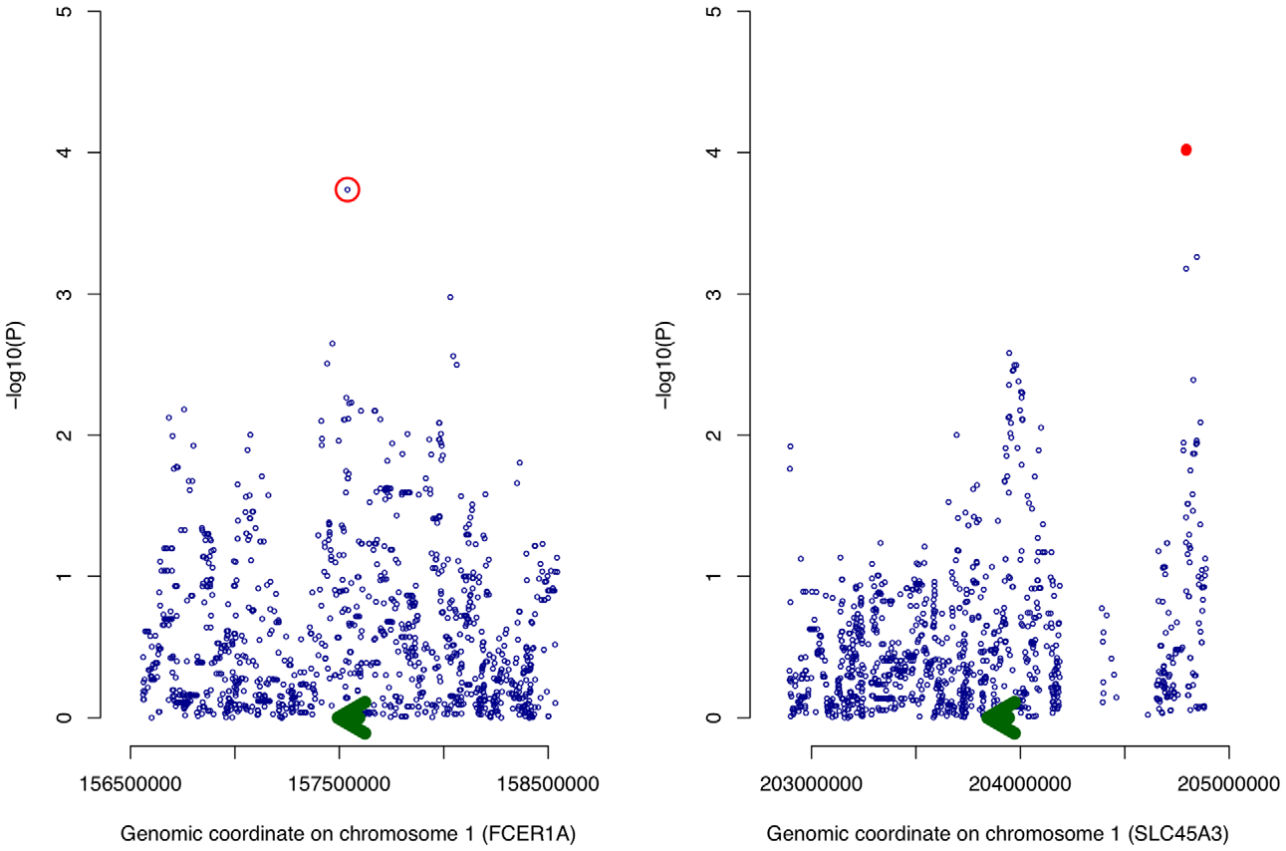
Association of genetic variation with expression of *FCER1A* and *SLC45A3*. For each *cis* SNP proximal to *FCER1A* and *SLC45A3*, a simple linear model is fitted and the regression *P* value (−log10 transformed) plotted along the vertical axis with genomic position of the SNP along the horizontal axis (both on chromosome 1). The two adjacent SNPs passing a permutation threshold of 0.05 are denoted by red dots while the SNP (rs2251746) within *FCER1A* found to drive expression of the LL module is circled in red. The position of the expression probe and the direction of transcription are denoted by a green arrow.

At a permutation threshold of 0.05, only two *cis* SNP associations associated with genes in LL module (**Table S3**), *SLC45A3* expression was associated with variation at rs12569123 and rs12569261, however there was insufficient evidence for either SNP's association with overall expression of the LL module (*P* = 0.18 and *P* = 0.057 respectively). It was of note that rs2251746, an experimentally verified eQTL of *FCER1A* and the strongest signal in a recent GWAS for serum IgE levels [34], [45], nominally influenced *FCER1A* expression (**Figure 4**, nominal *P* = 1.83×10^−4^). In testing association with LL module expression, rs2251746 showed strong evidence (*P* = 4.28×10^−6^). For *trans* SNPs, only three significant associations were observed, all between *MS4A3* expression and a haploblock on chromosome 6 containing *PNRC1* and *SRrp35*. These SNPs also strongly predicted LL module expression (**Table S4**). Overall, the strongest signal for LL module expression corresponded to rs2251746, evidence that LL module contains both transcriptional and genetic components of the immune response.

### Structural equation modeling shows Lipid Leukocyte module is reactive to lipids

**Figure 5:**
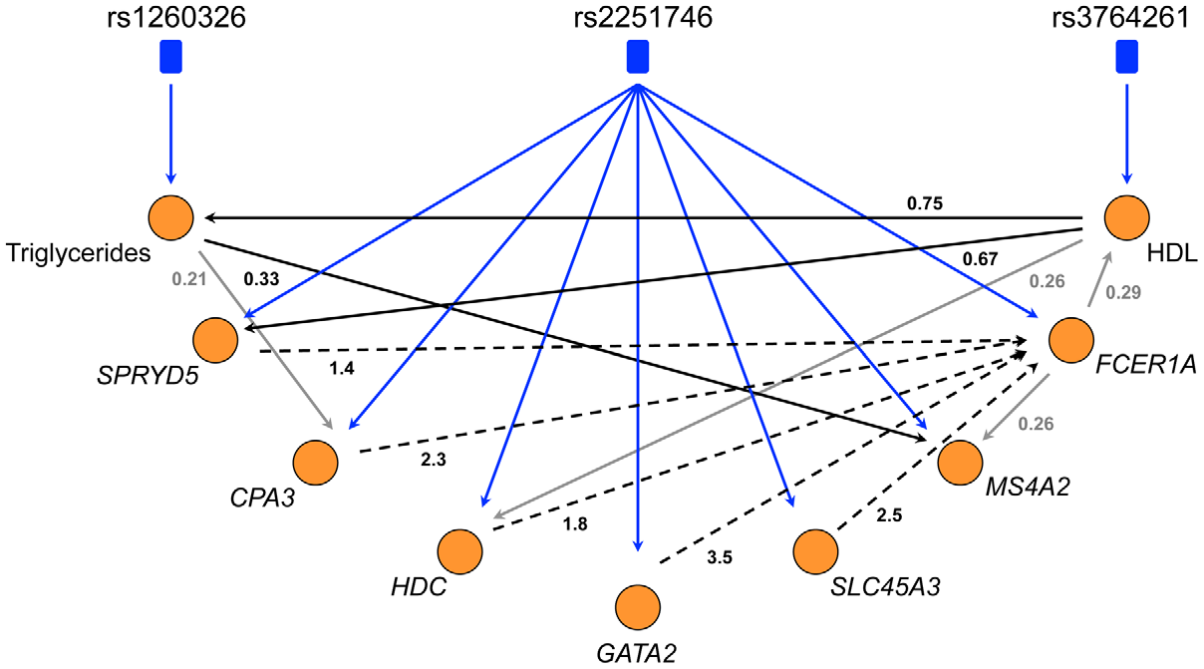
The directed network of core LL module, HDL, and triglycerides. NEO was used to generate an edge-oriented network of triglycerides, HDL, and core LL module genes. Blue edges denote significant correlations between SNPs and nodes (orange circles), black edges denote significant correlations between nodes with a corresponding LEO score greater than 0.3 (predicted as a causal edge) while grey edges denote significant correlations between nodes with a LEO score less than 0.3 (causality is inconclusive). Dotted edges signify causal model fitted *P* values <0.05.

Genetic variation can be used to orient network edges and infer causality [46]–[48]. Since we have identified genetic variation driving expression of LL module, we can construct a directed network of core LL module and other lipid measures which have been strongly associated with genetic variants (TG and HDL). To do this, we use SEM as implemented in Network Edge Orienting (NEO) methods [48]. A Local Edge Orienting (LEO) score was calculated to infer edge orientation (**Materials and Methods**). Simulation studies have previously shown that a LEO score threshold of 0.3 corresponds to a false positive rate less than 0.05 [48]. With this approach, we show that both HDL and TG may be causative of LL module by driving expression of *SPRYD5* (LEO score = 0.67) and *MS4A2* (LEO score = 0.33) respectively (**Figure 5**). Interestingly, HDL also appears to influence TG levels (LEO score = 0.75). In addition, core LL module genes were predicted to drive expression of *FCER1A* (minimum LEO score = 1.4) with the exception of *MS4A2*, high affinity IgE receptor's beta subunit. However, for these particular edges it should be noted that while deviation from the causal model was at least 25 times less likely than all other models considered, the causal model *P* values indicated that the causal model itself was likely a poor fit (**Table 7**). As more eQTLs are uncovered for LL module genes it is likely that model fitting will improve and the chain of causation within LL module will become clearer.

**Table 7:** LL core directed network statistics.

Edge (A→B)	LEO score (LEO.NB.OCA)	Model P value (A→B)	Pearson	Path Z statistic
HDL→TG	0.746	0.888	−0.34	−8.22
HDL→SPRYD5	0.671	0.466	0.19	4.52
TG→MS4A2	0.334	0.825	−0.45	−11.4
FCER1A→HDL	0.29	0.718	0.23	5.69
HDL→HDC	0.259	0.732	0.24	5.59
TG→CPA3	0.214	0.676	−0.49	−13
FCER1A→MS4A2	0.259	0.135	0.74	25.3
SPRYD5→FCER1A	1.41	1.35E-05	0.62	18.1
HDC→FCER1A	1.76	5.84E-06	0.7	22.2
CPA3→FCER1A	2.31	1.93E-06	0.71	23.1
SLC45A3→FCER1A	2.53	1.48E-06	0.66	20
GATA2→FCER1A	3.54	3.32E-08	0.67	20.3
MS4A2→HDL	−6.22	3.75E-07	0.17	3.87
GATA2→HDL	−6.29	5.15E-07	0.2	4.56
SLC45A3→HDL	−6.02	6.86E-07	0.22	5.15
CPA3→HDL	−6.06	6.85E-07	0.19	4.5
HDC→HDL	−5.8	1.17E-06	0.24	5.59
SPRYD5→HDL	−5.24	2.69E-06	0.19	4.52
MS4A2→FCER1A	−0.259	0.000297	0.74	25.3
FCER1A→GATA2	−4.15	4.33E-05	0.67	20.3
FCER1A→SLC45A3	−2.53	0.00245	0.66	20
FCER1A→CPA3	−2.31	0.00274	0.71	23.1
GATA2→TG	−2.23	0.00431	−0.45	−11.5
FCER1A→TG	−0.035	0.288	−0.45	−11.4
SLC45A3→TG	−2.22	0.00504	−0.46	−11.8
SPRYD5→TG	−2.1	0.00585	−0.4	−9.9
FCER1A→HDC	−1.76	0.00796	0.7	22.2
HDC→TG	−1.94	0.00887	−0.51	−13.6
FCER1A→SPRYD5	−1.41	0.0224	0.62	18.1
CPA3→TG	−1.48	0.0225	−0.49	−13
MS4A2→TG	−1.51	0.0255	−0.45	−11.4
TG→FCER1A	−0.224	0.186	−0.45	−11.4
TG→GATA2	−0.826	0.109	−0.45	−11.5
HDL→SLC45A3	−0.204	0.451	0.22	5.15
HDL→MS4A2	−0.553	0.175	0.17	3.87
TG→HDL	−0.884	0.116	−0.34	−7.85
TG→SPRYD5	−0.671	0.156	−0.4	−9.9
HDL→CPA3	−0.223	0.475	0.19	4.5
HDL→FCER1A	−0.616	0.174	0.23	5.34
TG→SLC45A3	−0.783	0.137	−0.46	−11.8
TG→HDC	−0.464	0.268	−0.51	−13.6
HDL→GATA2	−0.495	0.319	0.2	4.56

### Conclusions

In this report, a previously uncharacterized, potentially tissue-specific gene network (LL module) has been shown to be associated with blood lipid levels. The LL module not only harbors key components of inflammation and allergy which strongly suggest a role for basophils and mast cells but also associates with the SNP that most strongly regulates serum IgE levels. BMCs themselves have been previously associated with atherosclerosis and myocardial infarction [25], [29], [49], [50], however their precise role remains to be elucidated. The LL module described here offers genomic evidence in support of previous functional studies that LL module genes are linked to lipids and metabolism and, importantly, shows that these genes operate as a single gene module. This work provides a general framework to understand how lipid levels might activate cellular pathways in circulating nucleated peripheral blood cells contributing to cascades potentially resulting in atherosclerosis. Our findings should stimulate further, better-targeted molecular experiments to characterize details of this link.

## Materials and Methods

### Ethics statement

The DILGOM participants provided written informed consent. The protocol was designed and performed according to the principles of the Helsinki Declaration and was approved by the Coordinating Ethical Committee of the Helsinki and Uusimaa Hospital District.

### Trait measurements and sample collection

The study samples included a total of 631 unrelated Finnish individuals aged 25–74 years from the Helsinki area, recruited during 2007 as part of the Dietary, Lifestyle, and Genetic determinants of Obesity and Metabolic syndrome (DILGOM) study, an extension of the FINRISK 2007 study. Extensive trait information was collected, including lifestyle factors. Study participants were asked to fast overnight (at least 10 hours) prior to giving a blood sample. After extraction, the blood samples were left at room temperature for 45 minutes then centrifuged to separate the serum and plasma. Samples were kept in a −70°C freezer.

Total serum cholesterol (TC), high density lipoprotein cholesterol (HDL), low density lipoprotein cholesterol (LDL), apolipoprotein B (APOB), apolipoprotein A1 (APOA1), triglycerides (TG), and fasting free fatty acid (FFA) levels were determined in the Laboratory of Analytical Biochemistry of the Institute of Health and Welfare (Helsinki, Finland). TC measurements were carried out with the CHODPAP-assay (Abbott Laboratories, Abbott Park, Illinois, USA). HDL measurements used a direct enzymatic assay (Abbott Laboratories, Abbott Park, Illinois, USA). TG was measured with the enzymatic GPO assay (Abbott Laboratories, Abbott Park, Illinois, USA). APOB and APOA1 levels were determined using an immunoturbidometric method (Abbott Laboratories, Abbott Park, Illinois, USA). For APOB, the coefficients of variation (CVs) were 3.8%, 3.4% and 2.1% at the levels 0.35 g/L, 0.90 g/L and 1.66 g/L respectively. For APOA1, the CVs were 2.0%, 1.4% and 1.6% at the levels 0.91 g/L, 1.19 g/L and 2.15 g/L respectively. All methods used manufacturer protocols. FFA was determined using the enzymatic colorimetric ACS-ACOD method, as implemented in the NEFA-C assay kit, using the Architect c8000 (Abbott Laboratories, Abbott Park, Illinois, USA). Between series repeatability were 0.73 mmol/L, CV = 2.4% (n = 143) for level 1 and 0.99 mmol/L, CV = 2.3% (n = 139) for level 2. All methods used manufacturer protocols.

### Genotyping and expression microarrays

DNA was extracted from 10 ml EDTA whole blood samples with salt precipitation using Autopure (Qiagen GmbH, Hilden, Germany). DNA purity and quantity were assessed with PicoGreen (Invitrogen, Carlsbad, CA, USA). Genotyping used 250 ng of DNA and proceeded on the Illumina 610-Quad SNP array (Illumina Inc., San Diego, CA, USA) using standard protocols. After excluding chip failures and poor quality samples (as determined by visual inspection of a 0.75% agarose gel or low Sequenom call rate), 555 samples were successfully genotyped.

To obtain stabilized total RNA, we used the PAXgene Blood RNA System (PreAnalytiX GMbH, Hombrechtikon, Switzerland), which included collection of 2.5 ml peripheral blood into PAXgene Blood RNA Tubes (Becton Dickinson and Co., Franklin Lakes, NJ, USA) and total RNA extraction with PAXgene Blood RNA Kit (Qiagen GmbH, Hilden, Germany). We used the protocol as recommended by the manufacturer.

The integrity and quantity of the RNA sample was evaluated with the 2100 Bioanalyzer (Agilent Technologies, Santa Clara, CA, USA). Biotinylated cRNA was produced from 200 ng of total RNA with Ambion Illumina TotalPrep RNA Amplification Kit (Applied Biosystems, Foster City, CA, USA), using the protocol specified by the manufacturer. 750 ng of biotinylated cRNA were hybridized onto Illumina HumanHT-12 Expression BeadChips (Illumina Inc., San Diego, CA, USA), using standard protocol. For each sample, biotinylated cRNA preparation and hybridization onto BeadChip were done in duplicates. For expression arrays, 585 samples were successfully completed.

### Data quality, processing and imputation

After each expression array was scanned, background corrected probe signal intensities and bead counts were outputted from Illumina's BeadStudio software in order to undergo further processing. Strip-level quantile normalization was then used to force probe intensity distributions for all samples on all arrays to be the same [51]. Since each sample was technically replicated, the normalized values were then used to measure their correlation via Pearson's product moment correlation coefficient (Ρ) and Spearman's rank correlation coefficient (ρ). Generally, reproducibility was high (**Figure S2**). To further assess data quality, we also generated MA plots between replicate arrays after normalization [52]. We manually inspected each sample's MA plot for curvature or overt deviation from the M = 0 axis, none exhibited these characteristics. A sample was removed from further analysis if its Ρ was <0.94 or ρ was <0.60 (9 samples fail).

To combine raw signal intensities from corresponding replicates, the signals (*S*) were weighted by the number of beads (*b*) contributing to each signal and summed to arrive at one measure of signal intensity (δ) for each sample at each probe:
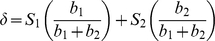
Probes that did not meet certain criteria were removed from further analysis: (a) non-autosomal (b) complementary to cDNA from erythrocyte globin components (c) map to more than one genomic position.

For each genotyping array, Cy3 and Cy5 signal intensities were exported from BeadStudio and pooled together for clustering with the Illuminus genotype calling algorithm [53]. Samples were removed from further analysis if they showed low quality (call rate <0.95, 19 samples removed), failed to match Sequenom genotype fingerprinting (concordance <0.90 for at least 10 genotypes, 0 samples removed), or were a previously unknown close relation or duplication (pairwise identity by descent pi-hat >0.10, 1 sample removed). SNPs failing to meet the following quality thresholds were also removed from further analysis: call rate >0.95, minor allele frequency >0.01, and Hardy-Weinberg equilibrium *P* value >1.0×10^−6^. 37,558 SNPs were removed in total.

Un-observed SNPs were imputed with the software *IMPUTE* version 5 using phased HapMap release 22 haplotypes from the CEU panel [54]. A genotype was assigned if its posterior probability was >0.95 or missing if not, and all SNPs underwent the same filtering as those above. 249,345 SNPs were removed in total, leaving 2,061,516 SNPs for further analysis.

### Population structure

To control for structure in the Finnish population, we used principal components analysis (PCA) on the genotypic data in order to identify outliers who descend from outside the Helsinki region (**Figure S3**). All SNPs underwent PCA with the *EIGENSOFT* software [55]; regression residuals involving the 2 previous SNPs were used as inputs to correct for SNP linkage disequilibrium. Samples exceeding eight standard deviations along any statistically significant principal component were removed from further analysis (**Figure S4**, 17 samples removed). A principal component was considered significant if its Tracy-Widom *P* value was <0.01.

### Trait distributions and correlations

Trait distributions and inter-trait correlations were also examined. Given well-known gender differences between many of the traits, distributions for males and females were treated separately. If a trait was not normally distributed as determined by an Anderson-Darling test (*P*<0.01), a Box-Cox power transformation was implemented to achieve normality and each trait measurement was converted to a *Z* score. The *Z* scores for males and females were then combined for further analyses. Inter-trait correlations were calculated via Spearman's rank correlation coefficient, see **Figure S1**.

### Association analysis and multiple test correction

All univariate statistical tests and permutations were done using PopGenomix, a C++ package developed in the Dermitzakis laboratory for the analysis of gene expression data. To test the association of a transcript's expression with a given SNP, linear regression was used. A simple model was constructed where *Y_i_* is the probe's log_2_-normalized expression for individual *i*, *X_i_* is the genotype of the individual at a given SNP (encoded as 0, 1, or 2 for the number of minor alleles), and ε_i_ is a normally distributed random variable with mean equal to zero and constant variance:
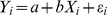
Nominal *P* values were calculated for the test of no association, *b* = 0.

In the case of Spearman's ρ, the coefficient is a function of ranks, *x_i_* is the rank of the log2-normalized expression value for individual *i*, *y_i_* is the genotypic rank (0, 1, or 2), and *n* is the corresponding number of measurements:
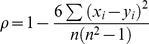
Since sample sizes were large, a *t*-test with *n*-2 degrees of freedom was used to determine a nominal *P* value.

Null distributions of *P* values were generated in order to evaluate the significance of the observed *P* value [43], [56], with expression levels permuted relative to genotypes. Unless otherwise specified, 10,000 permutations were performed, and each test was considered at an alpha level of 0.05.

Multiple and multivariate modeling was done using the R statistical computing language (http://www.r-project.org/). To test the association of a transcript's expression with a given trait, linear regression was used with appropriate covariates that include age, gender, or other correlated traits (see **Table 2**).

Given the highly correlated nature of the trait measurements, the construction of meta-traits was investigated. The meta-lipids (TC, FFA, HDL, LDL, TG, APOB, APOA1) trait was treated as the response variable in a multivariate linear model with probe expression, age, hypertension medication, and cholesterol medication as regressors (**Table 2**).
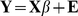
where **Y** is a matrix of normalized individual lipid trait values for genes; **X** is a matrix of log2-normalized expression values, age values, hypertension and cholesterol medication (as factors) for each individual; and **E** is a data matrix of error terms. Similarly, when testing SNP association with expression of all LL module genes simultaneously, a simple multivariate linear model was used. In which case **Y** is a matrix of log2-normalized individual expression values for genes in LL module, **X** is a vector of individual SNP genotypes (encoded 0, 1, 2), and **E** is a data matrix of error terms.

Reported *P* values are from the Wilks' lambda test statistic [57]. Multiple and multivariate modeling use the Bonferroni correction to control for multiple tests. All reported *P* values are corrected unless otherwise noted.

### Correction for cell type expression markers

To correct for relative cell type numbers, we use the expression markers defined in Whitney et al. [26]. The cell type proportions corrected for include lymphocytes, neutrophils, reticulocytes, B cells, cytotoxic T lymphocytes/natural killer cells, erythrocytes, myeloid cells, and Myc-regulated cells (profiles for the time of day were also included), however correction for T cells (uncovered on HT-12 array), mast cells (not assessed in Whitney et al.), and basophils specific markers (not assessed in Whitney et al.) was not possible. Covariates for the cell types were defined as the average standard score across all cell-type specific markers for each sample.

### Network analysis

Network analysis was done using the R packages, WGCNA [39], [40], [58] and NEO [48].

The undirected transcription network considered the top 10% of expression signals for meta-lipids (3,520 unique signals). The correlation matrix was constructed via all against all Pearson correlation coefficient calculations and the adjacency matrix was calculated with a soft threshold power of nine (**Figure S5**). Genes were then hierarchically clustered and visualized in a dendrogram (**Figure S6**), where a ‘leaf’ constitutes an individual gene and ‘branches’ are clusters of tightly correlated genes. The dynamic tree cut function in WGCNA with a minimum module size of 10 genes was used to determine initial modules. Individual module expression profiles underwent singular value decomposition and the summary module profiles from the vector corresponding to the first singular value were clustered to identify modules that were highly correlated (those less than a dendrogram height of 0.20). These modules were then merged.

To correlate module summary profiles with lipid traits, a *t*-test of Spearman's rank correlation was used. The corresponding Spearman correlation coefficients and *P* values can be observed in **Figure 1**. Statistical significance was determined by estimating the number of co-expression modules in the entire dataset. Given the 23 modules calculated from 1000 expression probes, we estimated the total number of modules to be (23×35419/1000) = 814.637. Therefore, the appropriate alpha level was determined to be (0.05/814.637) = 6.14×10^−5^. Calculations of module membership and individual gene significance (**Figure 3**) have been previously defined [39]. Only module K (the Lipid Leukocyte, LL, module) was used in further analyses.

NEO was used to predict the directedness of the network using causal SNPs as anchors. Of the lipid traits associated with LL module expression, HDL and TG were selected because the genetic variation underlying them has been studied extensively. Since the choice of SNPs can have a large impact on the directedness of the network (non-causal SNPs can introduce noise) and the DILGOM dataset (N = 518) is not sufficiently powered to significantly detect many of the known variants, we use only the strongest signals from recent genome-wide association studies [4], [59]; rs3764261 (*CETP*) was included for HDL and rs1260326 (*GCKR*) was included for TG. In our dataset, the strongest signal previously found for TG, rs964184 (*APOA1-C3-A4-A5*), did not pass quality control filters. Since rs2251746 has been shown to be an eQTL for *FCER1A* and LL module expression, we also include it as a causal anchor. To further verify that these loci can be considered causal anchors in the DILGOM dataset, we adopt the automatic SNP selection approach in NEO using both a greedy method and forward-stepwise regression [48]. We observed that all SNPs were correctly assigned to their respective nodes. An edge exists if the edge score (the absolute value of the Pearson correlation between nodes A and B) exceeds a threshold of 0.3. Since all nodes have a causal anchor, the NEO score (the log_10_ ratio of a fitted causal model *P* value to the next best causal model *P* value) defined in the main text is the NEO.NB.OCA score. An edge is considered significantly oriented if the NEO score exceeds a threshold of 0.3. Simulation studies have shown that a NEO.NB.OCA score of 0.3 or more corresponds to a false positive rate of 5% or less (cite NEO). We further considered the path coefficient for A→B (*Z* test statistic >1.96 or <−1.96) and, since the NEO score is a ratio of model *P* values, the fit of the primary model M_A_→A→B←M_B_ (*P* value should be >0.05). See **Table 7** for directed network edge statistics.

### Data availability

The expression data for the individuals analyzed in this study has been made publicly available through the ArrayExpress database (accession number E-TABM-1036).

## Supporting Information

Figure S1 Inter-trait correlations from the DILGOM population sample. Each tile is the color-coded Spearman rank correlation coefficient between any two trait measurements across the assessed DILGOM samples. Using the color bar on the right, a red tile indicates a strong positive correlation while a green tile indicates a strong negative correlation. No inter-trait correlation is signified by a white tile (the main diagonal is white by default).
(0.24 MB TIF)

Figure S2 Pearson and Spearman correlation coefficient distributions for technical replicates. Technical replicates of the Illumina HT-12 expression arrays displayed high reproducibility.
(0.13 MB TIF)

Figure S3 PCA of genotype data with no outliers removed. Principal components analysis was used to identify ethnically outlying samples.
(0.13 MB TIF)

Figure S4 PCA of genotype data after ethnic outlier removal. Seventeen samples were identified as ethnically differentiated from the rest of the DILGOM cohort (see Materials and Methods). After removal, the cohort shows no significant population structure.
(0.24 MB TIF)

Figure S5 Selection of adjacency matrix soft threshold power. To better differentiate strong and weak correlations and approximate scale-free network topology, each element of the expression correlation matrix is raised to a power β. Here, the selection of β follows the following criteria [58] (a) it maximizes the connectivity of network and (b) approximates scale-free network topology at a signed R^2^>0.80.
(0.15 MB TIF)

Figure S6 Transcription network dendrogram and module determination. Modules are determined via hierarchical clustering and dynamic branch cutting with a minimum module size of 10 genes. The module assignments are color-coded under ‘Dynamic Tree Cut’. Since initial branch cutting can produce modules which are themselves correlated with each other, a module merging step was implemented where all modules underwent singular value decompositions and were clustered [39]. The merged modules are color-coded under ‘Merged dynamic’. After merging, 23 modules were taken forward for further analysis.
(1.23 MB TIF)

Table S1 Genes showing significant evidence of association with lipid meta-trait.
(0.04 MB XLS)

Table S2 Genes comprising the LL module.
(0.02 MB XLS)

Table S3 Cis expression quantitative trait loci in the LL module.
(0.02 MB XLS)

Table S4 Trans expression quantitative trait loci in the LL module.
(0.02 MB XLS)

## References

[1] Kannel WB, Dawber TR, Kagan A, Revotskie N, Stokes J (1961). Factors of risk in the development of coronary heart disease–six year follow-up experience. the framingham study.. Ann Intern Med.

[2] Miller NE, Miller GJ (1975). Letter: High-density lipoprotein and atherosclerosis.. Lancet.

[3] Ross R (1999). Atherosclerosis–an inflammatory disease.. N Engl J Med.

[4] Aulchenko YS, Ripatti S, Lindqvist I, Boomsma D, Heid IM (2009). Loci influencing lipid levels and coronary heart disease risk in 16 european population cohorts.. Nat Genet.

[5] Prokopenko I, Langenberg C, Florez JC, Saxena R, Soranzo N (2009). Variants in MTNR1B influence fasting glucose levels.. Nat Genet.

[6] Sabatti C, Service SK, Hartikainen AL, Pouta A, Ripatti S (2009). Genome-wide association analysis of metabolic traits in a birth cohort from a founder population.. Nat Genet.

[7] Sandhu MS, Waterworth DM, Debenham SL, Wheeler E, Papadakis K (2008). LDL-cholesterol concentrations: A genome-wide association study.. Lancet.

[8] Hansson GK (2005). Inflammation, atherosclerosis, and coronary artery disease.. N Engl J Med.

[9] Hotamisligil GS (2006). Inflammation and metabolic disorders.. Nature.

[10] Hirosumi J, Tuncman G, Chang L, Gorgun CZ, Uysal KT (2002). A central role for JNK in obesity and insulin resistance.. Nature.

[11] Baud V, Liu ZG, Bennett B, Suzuki N, Xia Y (1999). Signaling by proinflammatory cytokines: Oligomerization of TRAF2 and TRAF6 is sufficient for JNK and IKK activation and target gene induction via an amino-terminal effector domain.. Genes Dev.

[12] Yu C, Chen Y, Cline GW, Zhang D, Zong H (2002). Mechanism by which fatty acids inhibit insulin activation of insulin receptor substrate-1 (IRS-1)-associated phosphatidylinositol 3-kinase activity in muscle.. J Biol Chem.

[13] Arkan MC, Hevener AL, Greten FR, Maeda S, Li ZW (2005). IKK-beta links inflammation to obesity-induced insulin resistance.. Nat Med.

[14] Yu C, Chen Y, Cline GW, Zhang D, Zong H (2002). Mechanism by which fatty acids inhibit insulin activation of insulin receptor substrate-1 (IRS-1)-associated phosphatidylinositol 3-kinase activity in muscle.. J Biol Chem.

[15] Perseghin G, Petersen K, Shulman GI (2003). Cellular mechanism of insulin resistance: Potential links with inflammation.. Int J Obes Relat Metab Disord.

[16] Alipour A, van Oostrom AJ, Izraeljan A, Verseyden C, Collins JM (2008). Leukocyte activation by triglyceride-rich lipoproteins.. Arterioscler Thromb Vasc Biol.

[17] Vaisar T, Pennathur S, Green PS, Gharib SA, Hoofnagle AN (2007). Shotgun proteomics implicates protease inhibition and complement activation in the antiinflammatory properties of HDL.. J Clin Invest.

[18] Chen Y, Zhu J, Lum PY, Yang X, Pinto S (2008). Variations in DNA elucidate molecular networks that cause disease.. Nature.

[19] Emilsson V, Thorleifsson G, Zhang B, Leonardson AS, Zink F (2008). Genetics of gene expression and its effect on disease.. Nature.

[20] Bremer J (1983). Carnitine–metabolism and functions.. Physiol Rev.

[21] Sugden MC (2003). PDK4: A factor in fatness?. Obes Res.

[22] Wolins NE, Brasaemle DL, Bickel PE (2006). A proposed model of fat packaging by exchangeable lipid droplet proteins.. FEBS Lett.

[23] Bildirici I, Roh CR, Schaiff WT, Lewkowski BM, Nelson DM (2003). The lipid droplet-associated protein adipophilin is expressed in human trophoblasts and is regulated by peroxisomal proliferator-activated receptor-gamma/retinoid X receptor.. J Clin Endocrinol Metab.

[24] Olsen RK, Olpin SE, Andresen BS, Miedzybrodzka ZH, Pourfarzam M (2007). ETFDH mutations as a major cause of riboflavin-responsive multiple acyl-CoA dehydrogenation deficiency.. Brain.

[25] McQueen MJ, Hawken S, Wang X, Ounpuu S, Sniderman A (2008). Lipids, lipoproteins, and apolipoproteins as risk markers of myocardial infarction in 52 countries (the INTERHEART study): A case-control study.. Lancet.

[26] Whitney AR, Diehn M, Popper SJ, Alizadeh AA, Boldrick JC (2003). Individuality and variation in gene expression patterns in human blood.. Proc Natl Acad Sci U S A.

[27] Allison DB, Thiel B, St Jean P, Elston RC, Infante MC (1998). Multiple phenotype modeling in gene-mapping studies of quantitative traits: Power advantages.. Am J Hum Genet.

[28] Ferreira MA, Purcell SM (2009). A multivariate test of association.. Bioinformatics.

[29] Tanimoto A, Sasaguri Y, Ohtsu H (2006). Histamine network in atherosclerosis.. Trends Cardiovasc Med.

[30] Gonen B, O'Donnell P, Post TJ, Quinn TJ, Schulman ES (1987). Very low density lipoproteins (VLDL) trigger the release of histamine from human basophils.. Biochim Biophys Acta.

[31] Jorgensen EA, Vogelsang TW, Knigge U, Watanabe T, Warberg J (2006). Increased susceptibility to diet-induced obesity in histamine-deficient mice.. Neuroendocrinology.

[32] Fulop AK, Foldes A, Buzas E, Hegyi K, Miklos IH (2003). Hyperleptinemia, visceral adiposity, and decreased glucose tolerance in mice with a targeted disruption of the histidine decarboxylase gene.. Endocrinology.

[33] Kraft S, Kinet JP (2007). New developments in FcepsilonRI regulation, function and inhibition.. Nat Rev Immunol.

[34] Weidinger S, Gieger C, Rodriguez E, Baurecht H, Mempel M (2008). Genome-wide scan on total serum IgE levels identifies FCER1A as novel susceptibility locus.. PLoS Genet.

[35] Paananen K, Kovanen PT (1994). Proteolysis and fusion of low density lipoprotein particles independently strengthen their binding to exocytosed mast cell granules.. J Biol Chem.

[36] Kokkonen JO, Vartiainen M, Kovanen PT (1986). Low density lipoprotein degradation by secretory granules of rat mast cells. sequential degradation of apolipoprotein B by granule chymase and carboxypeptidase A.. J Biol Chem.

[37] Pejler G, Knight SD, Henningsson F, Wernersson S (2009). Novel insights into the biological function of mast cell carboxypeptidase A.. Trends Immunol.

[38] Tsai FY, Orkin SH (1997). Transcription factor GATA-2 is required for proliferation/survival of early hematopoietic cells and mast cell formation, but not for erythroid and myeloid terminal differentiation.. Blood.

[39] Horvath S, Dong J (2008). Geometric interpretation of gene coexpression network analysis.. PLoS Comput Biol.

[40] Langfelder P, Horvath S (2008). WGCNA: An R package for weighted correlation network analysis.. BMC Bioinformatics.

[41] Dimas AS, Deutsch S, Stranger BE, Montgomery SB, Borel C (2009). Common regulatory variation impacts gene expression in a cell type-dependent manner.. Science.

[42] Stranger BE, Nica AC, Forrest MS, Dimas A, Bird CP (2007). Population genomics of human gene expression.. Nat Genet.

[43] Stranger BE, Forrest MS, Clark AG, Minichiello MJ, Deutsch S (2005). Genome-wide associations of gene expression variation in humans.. PLoS Genet.

[44] Goring HH, Curran JE, Johnson MP, Dyer TD, Charlesworth J (2007). Discovery of expression QTLs using large-scale transcriptional profiling in human lymphocytes.. Nat Genet.

[45] Hasegawa M, Nishiyama C, Nishiyama M, Akizawa Y, Mitsuishi K (2003). A novel -66T/C polymorphism in fc epsilon RI alpha-chain promoter affecting the transcription activity: Possible relationship to allergic diseases.. J Immunol.

[46] Schadt EE, Lamb J, Yang X, Zhu J, Edwards S (2005). An integrative genomics approach to infer causal associations between gene expression and disease.. Nat Genet.

[47] Li R, Tsaih SW, Shockley K, Stylianou IM, Wergedal J (2006). Structural model analysis of multiple quantitative traits.. PLoS Genet.

[48] Aten JE, Fuller TF, Lusis AJ, Horvath S (2008). Using genetic markers to orient the edges in quantitative trait networks: The NEO software.. BMC Syst Biol.

[49] Kaartinen M, Penttila A, Kovanen PT (1994). Accumulation of activated mast cells in the shoulder region of human coronary atheroma, the predilection site of atheromatous rupture.. Circulation.

[50] Kovanen PT, Kaartinen M, Paavonen T (1995). Infiltrates of activated mast cells at the site of coronary atheromatous erosion or rupture in myocardial infarction.. Circulation.

[51] Bolstad BM, Irizarry RA, Astrand M, Speed TP (2003). A comparison of normalization methods for high density oligonucleotide array data based on variance and bias.. Bioinformatics.

[52] Irizarry RA, Hobbs B, Collin F, Beazer-Barclay YD, Antonellis KJ (2003). Exploration, normalization, and summaries of high density oligonucleotide array probe level data.. Biostatistics.

[53] Teo YY, Inouye M, Small KS, Gwilliam R, Deloukas P (2007). A genotype calling algorithm for the illumina BeadArray platform.. Bioinformatics.

[54] Marchini J, Howie B, Myers S, McVean G, Donnelly P (2007). A new multipoint method for genome-wide association studies by imputation of genotypes.. Nat Genet.

[55] Patterson N, Price AL, Reich D (2006). Population structure and eigenanalysis.. PLoS Genet.

[56] Churchill GA, Doerge RW (1994). Empirical threshold values for quantitative trait mapping.. Genetics.

[57] Mardia KV (1979). Multivariate analysis.

[58] Zhang B, Horvath S (2005). A general framework for weighted gene co-expression network analysis.. Stat Appl Genet Mol Biol.

[59] Willer CJ, Sanna S, Jackson AU, Scuteri A, Bonnycastle LL (2008). Newly identified loci that influence lipid concentrations and risk of coronary artery disease.. Nat Genet.

[60] Freeman TC, Goldovsky L, Brosch M, van Dongen S, Maziere P (2007). Construction, visualisation, and clustering of transcription networks from microarray expression data.. PLoS Comput Biol.

