# Supplementary figures and images for: An Immune Response Network Associated with Blood Lipid Levels
Source: PLoS Genet. 2010 Sep 9;6(9):e1001113. doi: 10.1371/journal.pgen.1001113 (PMC2936545; doi:10.1371/journal.pgen.1001113)

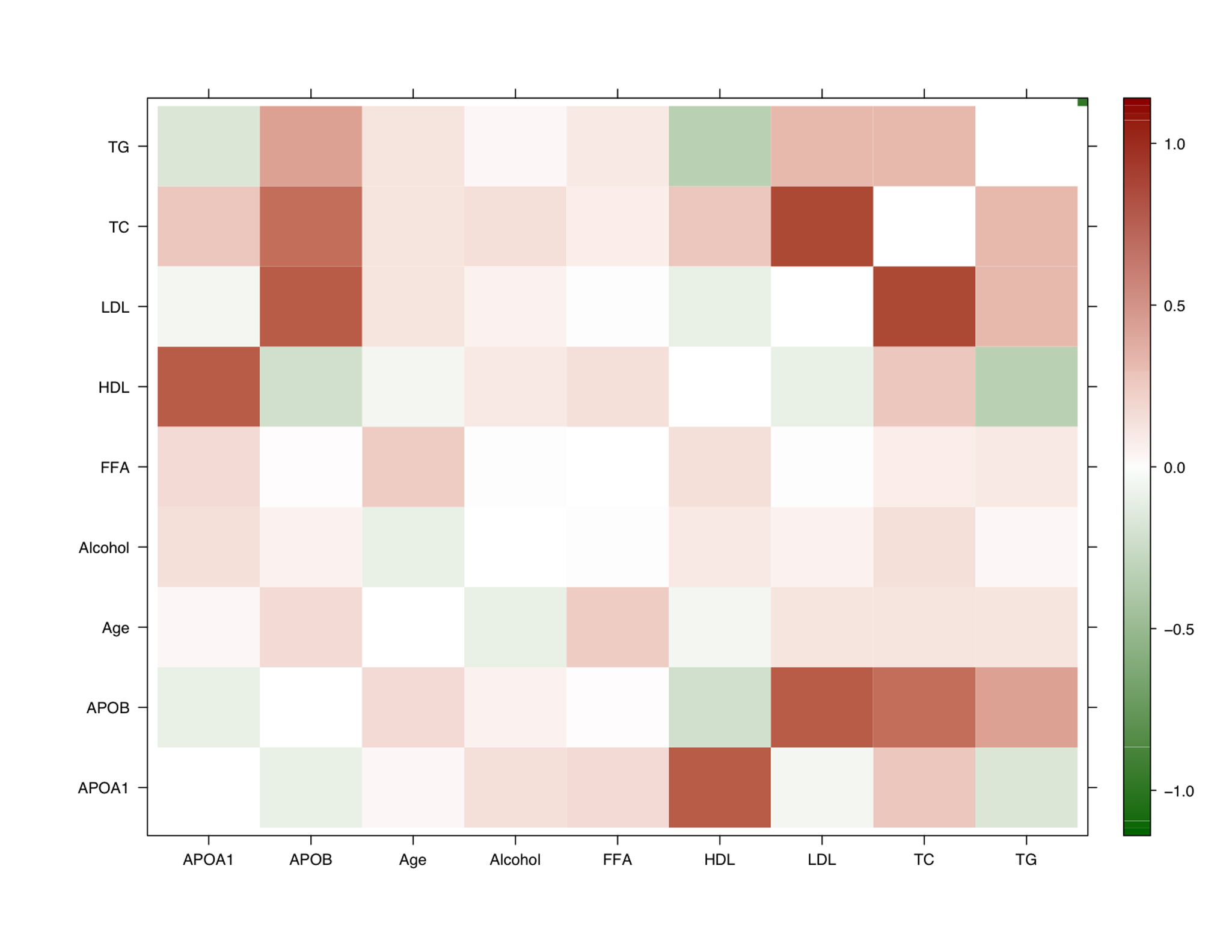

Supplement: Figure S1 — Inter-trait correlations from the DILGOM population sample. Each tile is the color-coded Spearman rank correlation coefficient between any two trait measurements across the assessed DILGOM samples. Using the color bar on the right, a red tile indicates a strong positive correlation while a green tile indicates a strong negative correlation. No inter-trait correlation is signified by a white tile (the main diagonal is white by default). (0.24 MB TIF) [file pgen.1001113.s001.tif]

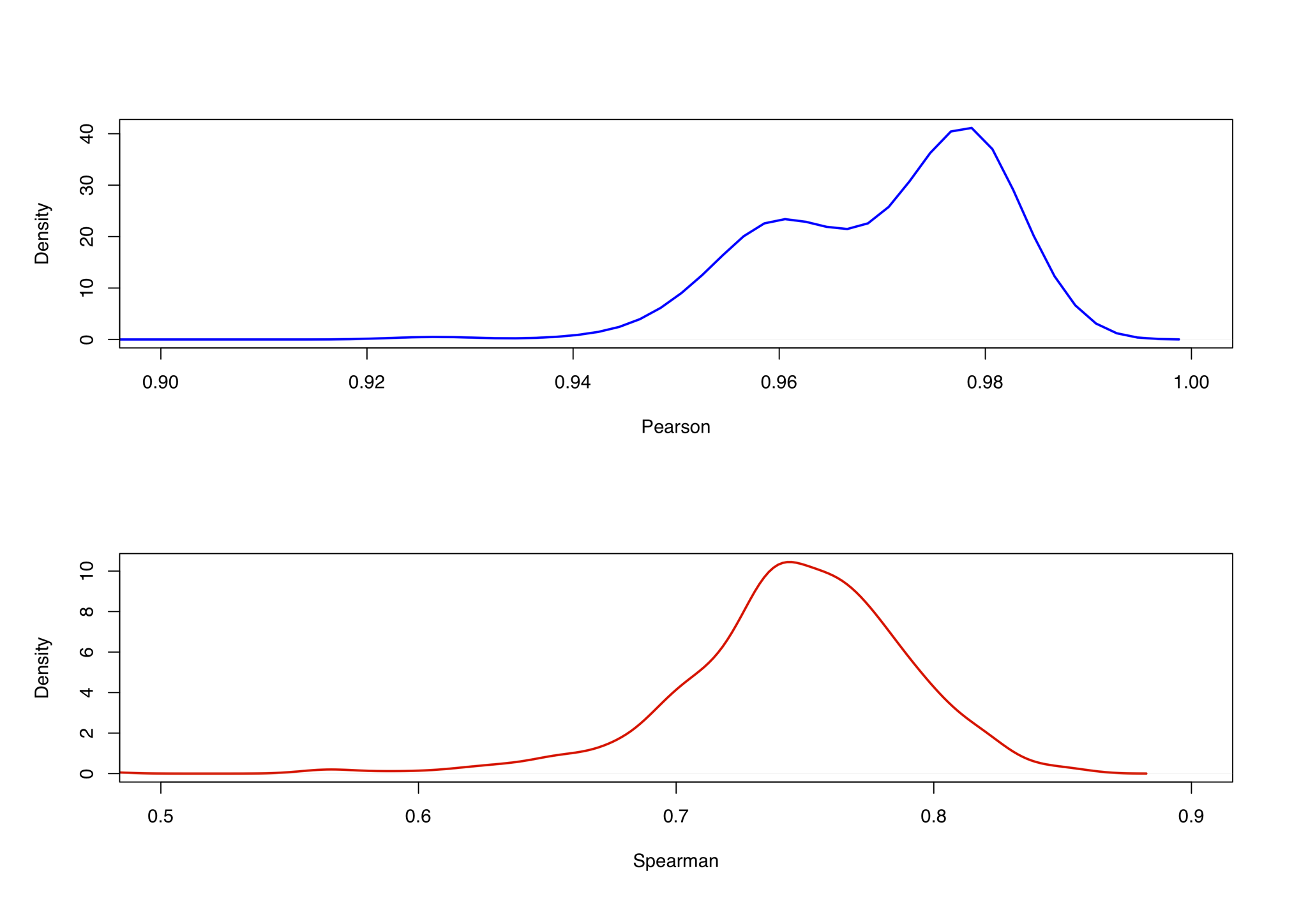

Supplement: Figure S2 — Pearson and Spearman correlation coefficient distributions for technical replicates. Technical replicates of the Illumina HT-12 expression arrays displayed high reproducibility. (0.13 MB TIF) [file pgen.1001113.s002.tif]

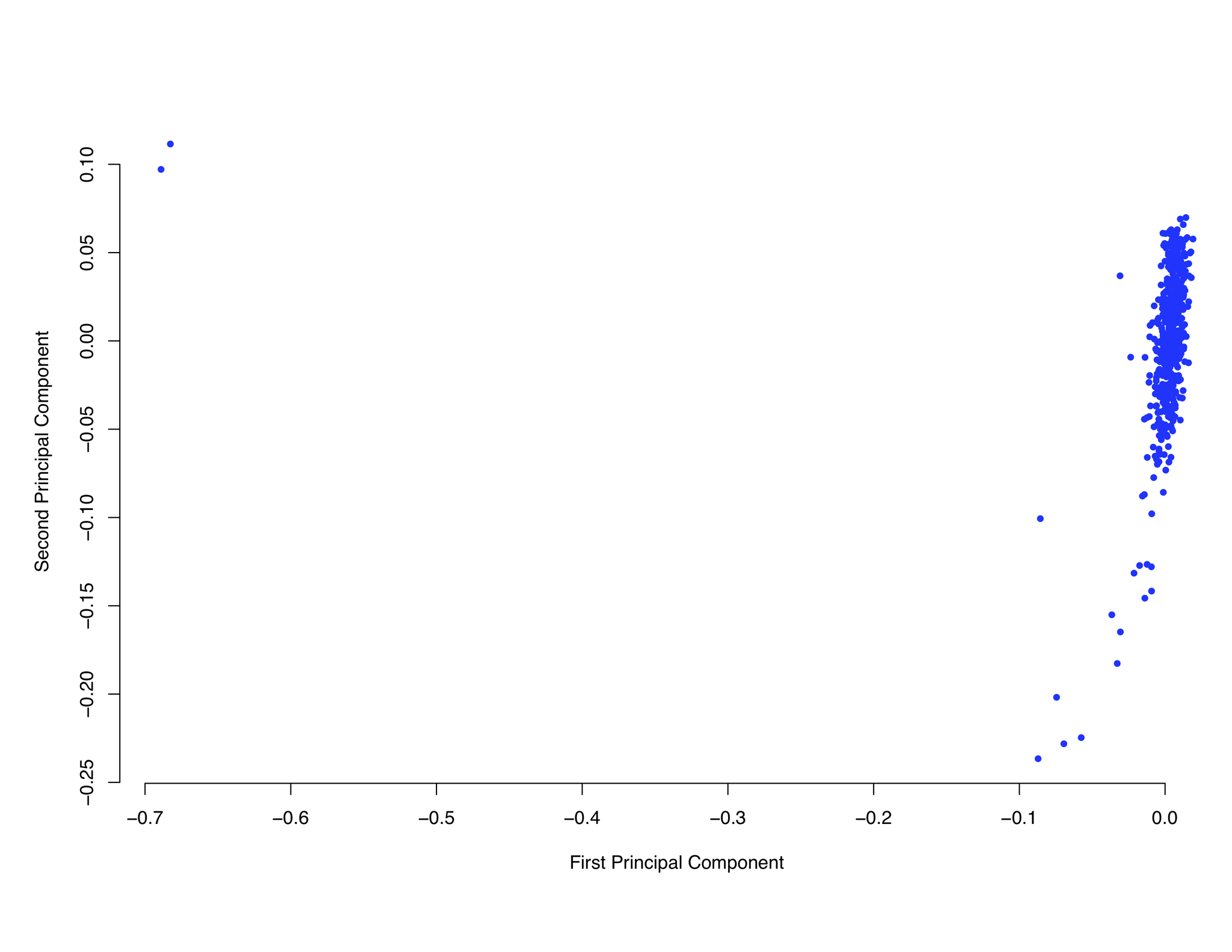

Supplement: Figure S3 — PCA of genotype data with no outliers removed. Principal components analysis was used to identify ethnically outlying samples. (0.13 MB TIF) [file pgen.1001113.s003.tif]

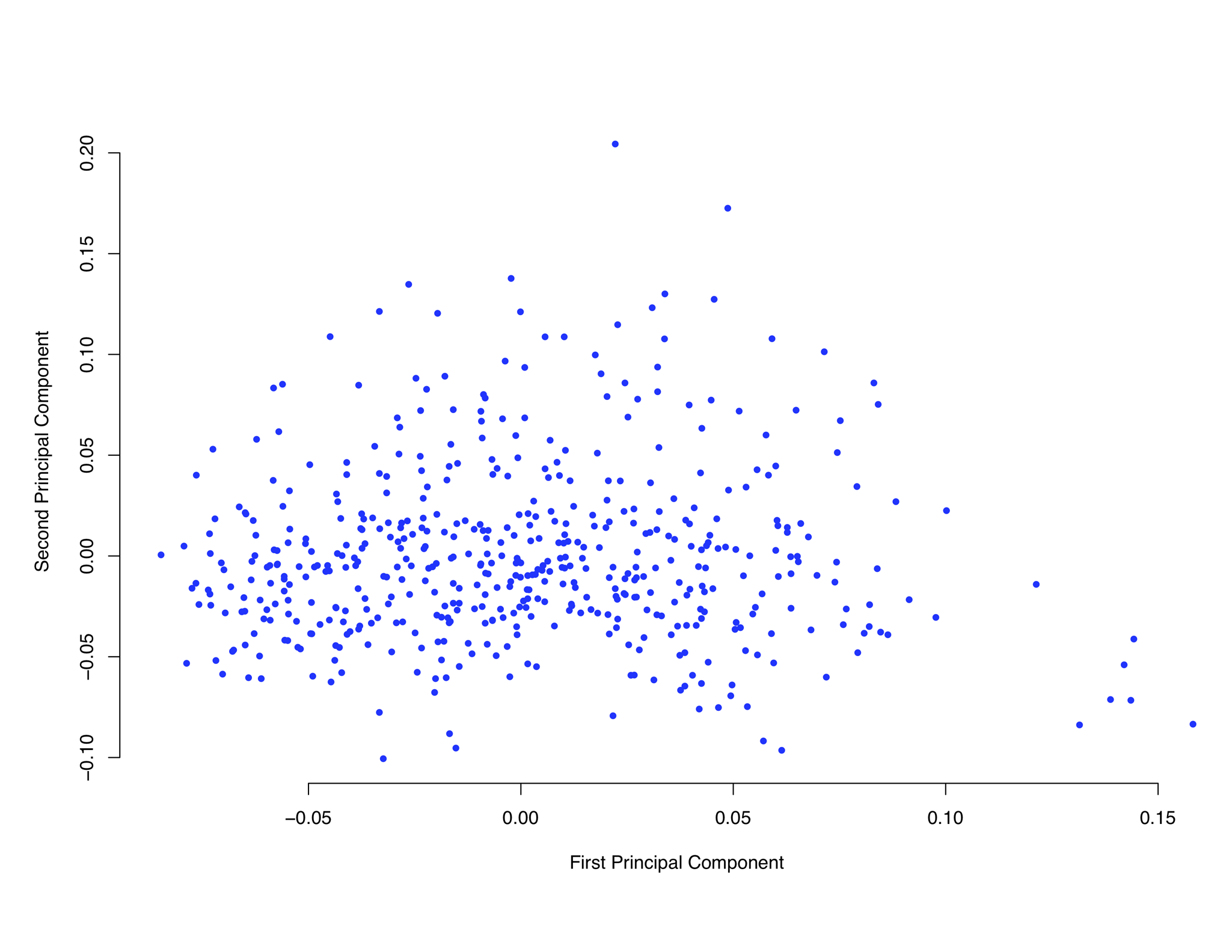

Supplement: Figure S4 — PCA of genotype data after ethnic outlier removal. Seventeen samples were identified as ethnically differentiated from the rest of the DILGOM cohort (see Materials and Methods). After removal, the cohort shows no significant population structure. (0.24 MB TIF) [file pgen.1001113.s004.tif]

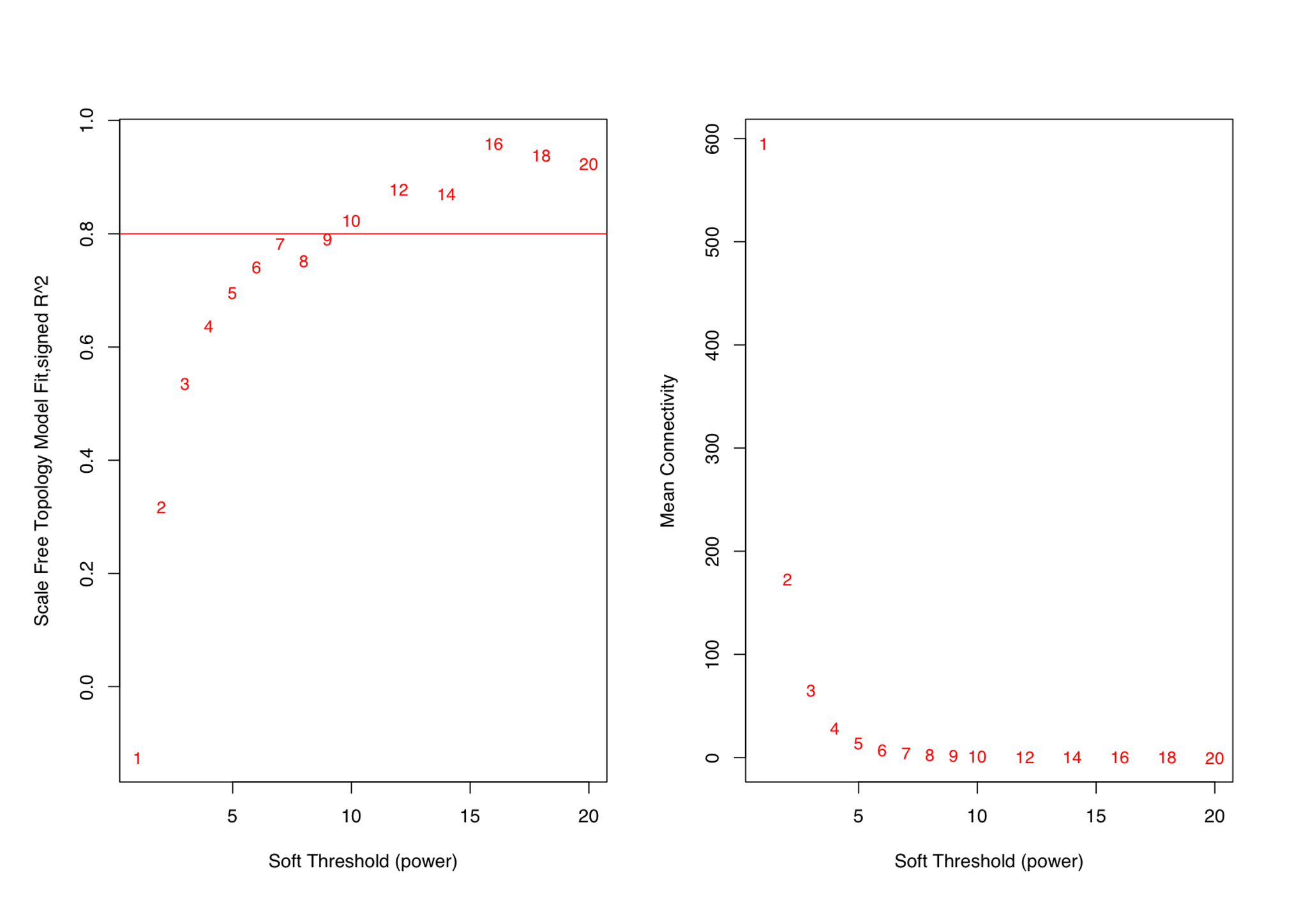

Supplement: Figure S5 — Selection of adjacency matrix soft threshold power. To better differentiate strong and weak correlations and approximate scale-free network topology, each element of the expression correlation matrix is raised to a power β. Here, the selection of β follows the following criteria [58] (a) it maximizes the connectivity of network and (b) approximates scale-free network topology at a signed R2>0.80. (0.15 MB TIF) [file pgen.1001113.s005.tif]

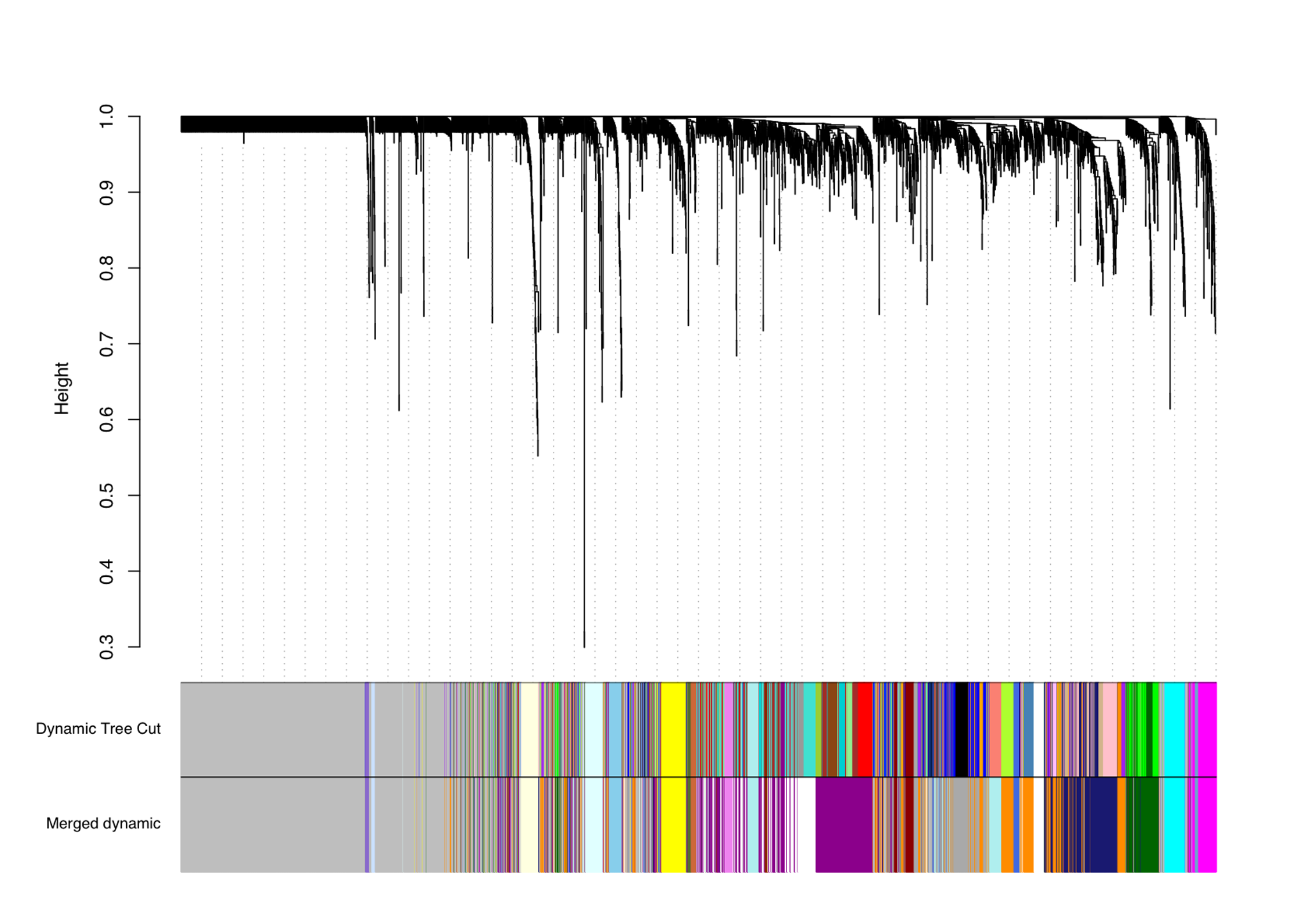

Supplement: Figure S6 — Transcription network dendrogram and module determination. Modules are determined via hierarchical clustering and dynamic branch cutting with a minimum module size of 10 genes. The module assignments are color-coded under ‘Dynamic Tree Cut’. Since initial branch cutting can produce modules which are themselves correlated with each other, a module merging step was implemented where all modules underwent singular value decompositions and were clustered [39]. The merged modules are color-coded under ‘Merged dynamic’. After merging, 23 modules were taken forward for further analysis. (1.23 MB TIF) [file pgen.1001113.s006.tif]
